# Diversified alternaria pathogenicity alters plant–soil feedbacks through leaf–root-microbiome dynamics in agroforestry systems

**DOI:** 10.1093/hr/uhaf137

**Published:** 2025-05-21

**Authors:** Lifen Luo, Zhengping Wang, Xiubei Yan, Chen Ye, Jianjun Hao, Xili Liu, Shusheng Zhu, Min Yang

**Affiliations:** State Key Laboratory for Crop Stress Resistance and High-Efficiency Production, College of Plant Protection, Northwest A&F University, Yangling 712100, China; State Key Laboratory for Conservation and Utilization of Bio-Resources in Yunnan, College of Plant Protection, Yunnan Agricultural University, Kunming 650201, China; State Key Laboratory for Conservation and Utilization of Bio-Resources in Yunnan, College of Plant Protection, Yunnan Agricultural University, Kunming 650201, China; State Key Laboratory for Conservation and Utilization of Bio-Resources in Yunnan, College of Plant Protection, Yunnan Agricultural University, Kunming 650201, China; State Key Laboratory for Conservation and Utilization of Bio-Resources in Yunnan, College of Plant Protection, Yunnan Agricultural University, Kunming 650201, China; School of Food and Agriculture, University of Maine, Orono, ME 04469, USA; State Key Laboratory for Crop Stress Resistance and High-Efficiency Production, College of Plant Protection, Northwest A&F University, Yangling 712100, China; State Key Laboratory for Conservation and Utilization of Bio-Resources in Yunnan, College of Plant Protection, Yunnan Agricultural University, Kunming 650201, China; State Key Laboratory for Conservation and Utilization of Bio-Resources in Yunnan, College of Plant Protection, Yunnan Agricultural University, Kunming 650201, China

## Abstract

Interspecific interactions including plant–plant, plant–microbe, and plant–insect are the important elements to drive the positive plant–soil feedback for maintaining ecosystem stability in biodiversity ecosystems. Yet, the role of diversified foliar pathogens in biodiversity system in influencing the plant–soil feedback (PSF) has often been underestimated. Here, we assessed the effects of foliar *Alternaria panax* pathogenicity diversity from agroforestry system on PSF and rhizosphere microbial community. We show that a moderate intensity of foliar pathogen infection by *A. panax* could activate jasmonic acid (JA)-mediated defense from shoots to roots. This activation enhanced the synthesis and secretion of 2-aminoethanesulfonic acid into the rhizosphere for a disease-suppressive rhizo-microbiota assembly, contributing to positive PSF. However, excessive foliar pathogen infection allocated JA-mediated defense only in leaf and disrupted this rhizomicrobial enrichment, resulting negative PSF. This study enhances the understanding of the ecological roles of foliar pathogen within agroforestry systems and provides an insight into agricultural sustainability.

## Introduction

Plant–soil feedbacks (PSF) refers to the bidirectional interaction between plants and soil, where plants alter soil biotic and abiotic components through chemical secretions, influencing subsequent plant growth in the same environment [1]. The root-associated microbiome is crucial in this process, affecting plant performance, ultimately determining whether PSF is positive, negative, or neutral [2]. Negative PSF can reduce soil suitability for the same species [3], playing a role in maintaining plant diversity and driving community dynamics in natural ecosystems [4, 5]. However, it also creates a hostile environment for subsequent crops, limiting sustainable productivity in intensive monocropping systems [6]. On the other hand, positive PSF enhances crop sustainability by providing a beneficial legacy for subsequent crops [2, 7]. Therefore, understanding how to maintain positive PSF is essential for sustainable agriculture.

Agroforestry systems, characterized by high biodiversity and multi-trophic interactions (e.g. plant–plant, plant–microbe), exhibit stabilized positive PSF through pathogen dilution and functional microbiome assembly [8–10]. These biodiversity systems harbor higher pathogen diversity and broader pathogenicity ranges compared to agricultural systems [11, 12], yet paradoxically maintain lower disease severity [13]. The varying virulence among strains and the intensities of pathogenicity within pathogen–environment–host interactions are well documented in agroforestry systems [12, 14]. However, their impact on PSF is not fully understood. Understanding the diverse interactions between pathogens and plants—particularly the effects of infection by pathogens with varying virulence levels and plant disease severity on positive PSF within diverse ecosystems—is critical for developing innovative disease management strategies.

Emerging evidence reveals that foliar pathogen attacks can trigger systemic immune responses, stimulating plants to secrete root metabolites that recruits beneficial rhizosphere microorganisms-a phenomenon described as the ‘cry for help’ strategy [15–17]. Our field observations in *P. notoginseng*-pine agroforestry systems align with this phenomenon: despite causing premature leaf abscission, leaf spot pathogen (*Alternaria* spp.) infections are followed by a high seedling emergence rate and positive PSF in subsequent years, especially in soil conditioned by mild disease. This implies that the diversified pathogenicity of *Alternaria* spp. may steer PSF directionality, potentially through establishing an ‘immuno-ecological memory’ in soils—a legacy of plant ‘cry for help’ strategy under biodiversity regimes, yet the regulatory mechanisms remain elusive.

Upon plant infection, immune responses engage pathogen-associated molecular pattern (PAMP)-triggered immunity (PTI) and effector-triggered immunity (ETI) [18]. A variety of key processes, including those mediated by salicylic acid (SA) and jasmonic acid (JA), play a key role in launching the plant immune signaling network following PTI or ETI [19]. These immune responses lead to alterations in the plant metabolism and root exudate composition, thereby influencing the rhizosphere microbiome [20–23]. Nevertheless, it remains unclear how foliar infections by *Alternaria* spp. with varying pathogenicity levels or intensities in agroforestry systems trigger the plant’s ‘cry for help’ response to shape soil ‘immuno-ecological memory’. Deciphering these mechanistic links—spanning pathogen-induced immunity, rhizosphere microbiome assembly, and PSF stability—will advance targeted design of microbiome-mediated strategies for sustainable crop protection.

The *Panax* genus, part of the Araliaceae family, is known for its medicinal value but is susceptible to soilborne pathogens, leading to strong negative PSF [24]. We have previously demonstrated that foliar infection of *Panax notoginseng* by *A. panax* shifts plant–soil interactions from negative to positive [5], but the instability of this shift underscores unresolved mechanistic complexities. We hypothesize that the directional outcomes in the rhizosphere soil are associated with the pathogenicity levels and intensity of foliar infection, mediated through root exudates–microbiome interactions. This study aims to examine how *Alternaria* isolates with varying pathogenicity or infection intensity drive PSF dynamics and to analyze the relationship between the PSF shifts and the changes in microbial community structure and function mediated by plant metabolic responses. We expect this research will elucidate the ecological role of pathogen infestation in regulating PSF within agroforestry systems, providing valuable insights into sustainable agricultural practices.

## Results

### Pathogenicity diversify of *A. panax* affects the direction of plant–soil feedback

**Figure 1:**
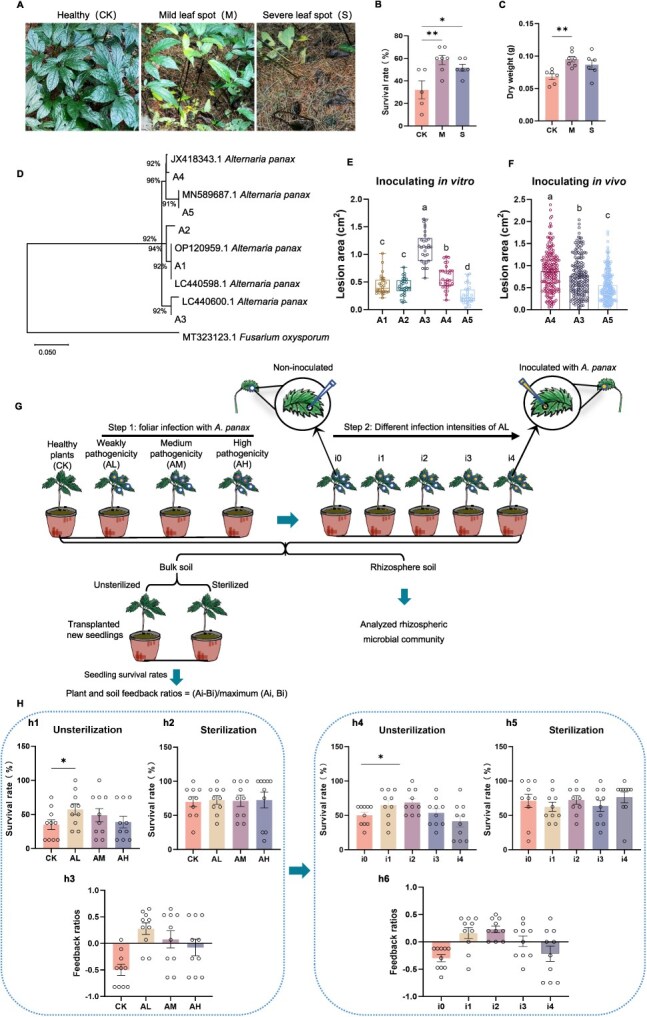
Impact of *A. panax* pathogenicity diversify on plant–soil feedback in an agroforestry system. (A) Performance of *P. notoginseng* under pine forests: healthy (CK), mild leaf spot with partial defoliation (M), and severe leaf spot with complete leaf loss (S). (B-C) Leaf infection effects on subsequent *P. notoginseng* survival rate (B) and dry weight (C) (*n* = 5, 7, 6 to assess the survival rate; *n* = 6, 7, 6 to evaluate dry weight). (D-F) Identification (D), and pathogenicity testing of *A. panax* on *P. notoginseng* leaves *in vitro* (E) (*n* = 30) and *in vivo* (F) (*n* = 183). CK indicates the blank control with a noncolonized agar block. (G) Experimental procedure overview: evaluating *A. panax*'s pathogenicity levels (weakly, medium, and high) and infection intensity (inoculated zero-, one-, two-, three-, or four leaf) on PSF and rhizosphere microbiome. *P. notoginseng* inoculated with *A. panax* strains AL (weakly), AM (medium), and AH (high) in step 1; noninoculated (i0) or inoculated with varying leaf numbers (i1–i4) in step 2. (H) Seedling survival in unsterilized and sterilized soils following foliar infection by *A. panax* at different pathogenicity levels (h1 and h2) or with the weakly pathogenic strain AL at varying intensities (h4 and h5) (n = 10). CK denotes healthy plants. (h3 and h6) Feedback ratios based on seedling survival rates in unsterilized soil following foliar infection by *A. panax* at different pathogenicity levels (h3) or strain AL at varying intensities (h6). CK compares unsterilized to sterilized soil feedback ratios. Data presented as mean ± SEM. Asterisks denote significant differences compared with blank control (two-tailed *t*-test, ^*^P < 0.05, **P < 0.05 -). Letters indicate significant differences (ANOVA with Tukey’s HSD, *P* < 0.05).

The subsequent growth in the soils amend with the rhizosphere soil of *P. notoginseng* with mild and severe leaf spot from an agroforestry system was analyzed (Fig. 1A–C). The survival rate and dry biomass of subsequent *P. notoginseng* were enhanced, particularly in soil conditioned by mild Alternaria leaf spot (Fig. 1B and C). Five strains of *A. panax,* isolated from the diseased leave of *P. notoginseng*, exhibited varying degrees of pathogenicity under *in vitro* inoculation conditions (Fig. 1D and E). Three strains exhibiting low, medium, and high levels of pathogenicity were selected for *in vivo* inoculation, and the results corroborated the differences in pathogenicity among the strains (Fig. 1F). Thus, we employed three strains of *A. panax* (A5, A3, and A4) to evaluate the effects of foliar infection by *A. panax* with low (AL), medium (AM), and high (AH) levels of pathogenicity on plant–soil feedback (PSF) (Fig. 1G). Our finding revealed that the rhizosphere soil from *P. notoginseng* without *A. panax* infection showed a suppressed offspring survival rate compared to sterilized soil, indicating negative PSF (Fig. 1h1-h3). However, when *P. notoginseng* leaves were inoculated with the weakly pathogenic strain AL, the survival rate of subsequent *P. notoginseng* significantly increased, which indicated positive PSF (Fig. 1h1 and h3). Yet, the survival rates declined in the soil where *P. notoginseng* had been inoculated with *A. panax* strains exhibiting medium (AM) and high (AH) levels of pathogenicity, which did not result in positive PSF compared to AL-conditioned plantation (Fig. 1h1 and h3).

To eliminate variables associated with strains with different levels of pathogenicity, we employed the weakly pathogenic strain AL for a focused study on the effects of infection intensity on PSF (Fig. 1G). Results demonstrated that a moderate infection intensity (inoculation of one- or two-leaf) significantly enhanced the survival rate of *P. notoginseng* plants in subsequent soil, resulting in positive PSF (Fig. 1h4 and h6). As infection intensity increased (inoculation of three- or four-leaf), the survival rate in subsequent soil decreased progressively (Fig. 1h4). Soil cultivated with *P. notoginseng* inoculated on four-leaf exhibited negative PSF (Fig. 1h6). However, these effects were not prominent after soil sterilization (Fig. 1h2 and h5). These findings indicate that foliar infection by a weak pathogenic strain of *A. panax* or at a moderate infection intensity induces the development of disease-suppressive soil, resulting in positive PSF.

### Foliar moderate infection enriched disease-suppressive microbiota responsible for positive PSF

**Figure 2:**
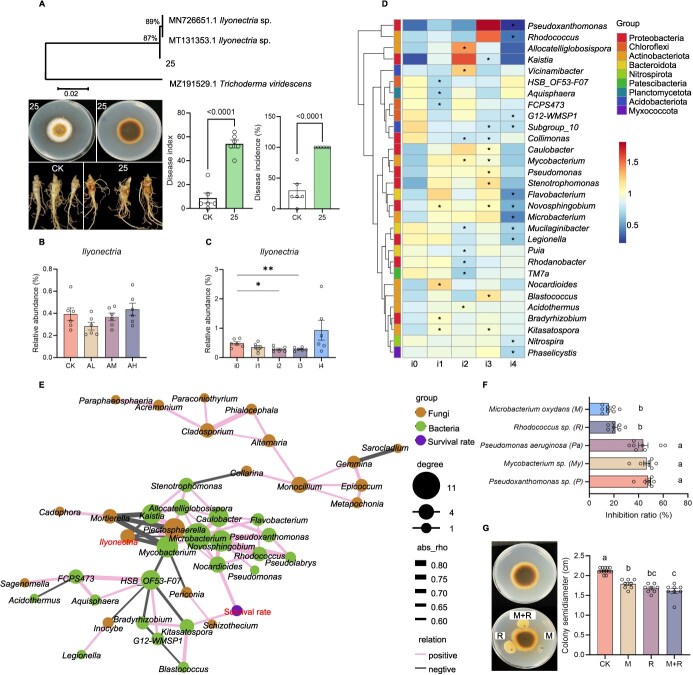
Effects of *A. panax* infections with varying pathogenicity (weakly, medium, high) and intensities (inoculated zero-, one-, two-, three-, or four leaf) on *P. notoginseng* rhizosphere microbial communities and functions. (A) Identification and pathogenicity of *Ilyonectria* sp. on *P. notoginseng* roots (*n* = 6). CK indicates the blank control with a noncolonized agar block. (B and C) Relative abundance shifts of *Ilyonectria* after foliar infection by *A. panax* with different pathogenicity (B) and intensities (C) (*n* = 6). CK indicates the blank control with healthy plants. (D) Top 30 genera shift in differential bacteria after foliar infection by *A. panax* with varied intensities (*n* = 6). i0, i1, i2, i3, and i4 denote the number of leaves infected by *A. panax* AL. (E) Correlation between *P. notoginseng* seedling survival and differential microbial populations after foliar infection by *A. panax*. (F) Antagonistic activity of distinct bacterial species against *Ilyonectria* sp. (*n* = 8). (G) Antagonistic activity of *Rhodococcus* sp. and *Microbacterium oxydans* mixture against *Ilyonectria* sp. (*n* = 13, 8, 8, 8). CK denotes the blank control with only *Ilyonectria* sp. grown. Data presented as mean ± SEM. Asterisks denote significant differences compared with blank control (two-tailed *t*-test, ^*^P < 0.05; ^**^P < 0.01); letters indicate significant differences (ANOVA with Tukey’s HSD, *P* < 0.05).

To explore the influence of pathogenic diversity of *A. panax* on the microbial communities in rhizosphere soils, samples were collected from *P. notoginseng* rhizosphere soil after foliar infection by *A. panax* with different pathogenicity and intensities. Principal coordinate analysis (PCoA) based on operational taxonomic units (OTUs) detected shifts in fungal and bacterial community compositions affected by different pathogenic strains’ infections (*P* = 0.004 for fungi; *P* = 0.04 for bacteria) and different infection intensities (*P* = 0.002 for fungi; *P* = 0.139 for bacteria) (Fig. S1). Fifty-one fungal genera (Table S1) and 73 bacterial genera (Table S2) were significantly influenced by the infection with strains of varying pathogenicity levels. Furthermore, 78 fungal genera (Table S3) and 100 bacterial genera (Table S4) were significantly altered by various infection intensities of *A. panax* AL. Random forest analysis identified *Ilyonectria* (asexual: *Cylindrocarpon*) and *Plectosphaerella* as the co-regulated biomarkers across the infections by both strains with different pathogenicity levels and varying infection intensities of AL, ranking among the top 15 biomarkers (Fig. S2A and B). *Ilyonectria* sp. were successfully isolated from diseased *P. notoginseng* roots and confirmed as a root rot pathogen (Fig. 2A). In the rhizosphere soil, the relative abundance of *Ilyonectria* decreased following foliar infection by the weakly pathogenic strain AL and by AL with two- and three leaf but increased after foliar infection by the highly pathogenic strain AH and by AL with four leaf (Fig. 2B and C).

**Figure 3:**
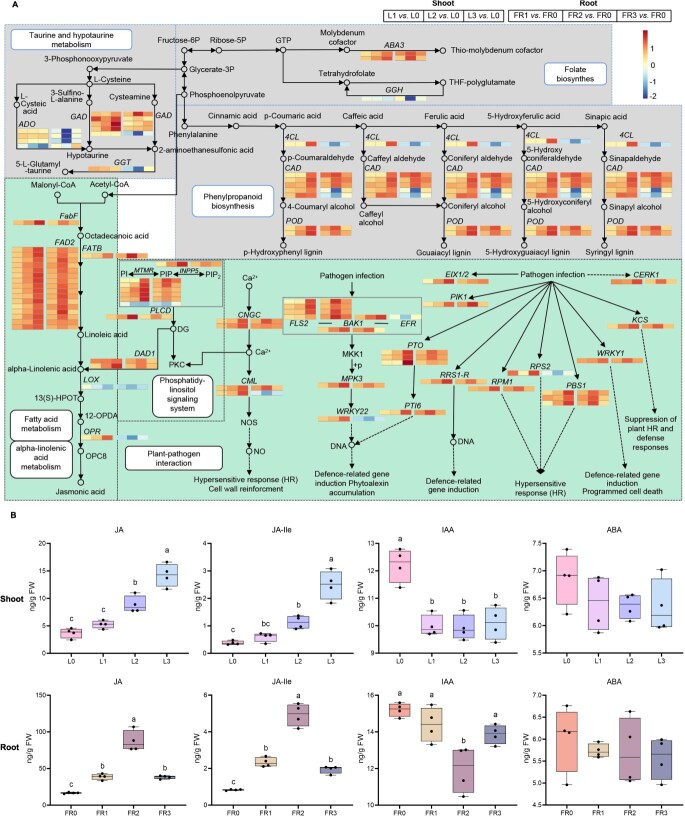
The weakly pathogenic strain AL infection with varying intensities (zero-, one-, two-, or three leaf) on *P. notoginseng* leaves affects defense responses in both shoots and fibrous roots. (A) Gene expression profiles for co-enriched defense pathways. (B) Hormone changes. FR0-FR3 corresponds to fibrous roots with 0–3 inoculated leaves (L0–L3). Letters indicate significant differences (ANOVA with Tukey’s HSD, *P* < 0.05, *n* = 4).

Among the top 30 differential bacterial genera after foliar infection by *A. panax* with different levels of pathogenicity, 18 potentially beneficial bacteria, including *Microbacterium*, *Mycobacterium, Pseudoxanthomonas, Pseudomonas, Rhodococcus, Allocatelliglobosispora, Flavobacterium,* and *Nocardioides*, were significantly enriched following infection by the weakly pathogenic strain AL (Fig. S2C). These genes’ enrichment was also observed after infection with one-, two-, and three leaves by *A. panax* AL but suppressed after infection with four leaf (Fig. 2D). These genera exhibited a negative correlation with *Ilyonectria* but a positive correlation with the survival rate of *P. notoginseng* seedlings (Fig. 2E). Notably, specific taxa such as *Pseudoxanthomonas* sp., *Mycobacterium* sp., *Pseudomonas aeruginosa*, *Rhodococcus* sp., and *Microbacterium oxydans* significantly inhibited the mycelial growth of *Ilyonectria* sp. (Fig. 2F). Although *Rhodococcus* sp. and *Microbacterium oxydans* exhibited lower individual antagonistic activity against *Ilyonectria* sp., their combined effect resulted in an enhanced antagonistic response, highlighting the synergistic potential of the core rhizosphere microbiota against *Ilyonectria* sp. (Fig. 2F and G).

### Foliar pathogen infection intensity determines the JA-mediated defense from shoots to roots

To analyze the defense response from shoots to roots affected by varying intensities of foliar pathogen infection, we conducted transcriptome sequencing after foliar infection with the AL strain on either zero-, one-, two-, or three leaf. PCA analysis showed that foliar infection by *A. panax* at different intensities led to alterations in gene expression profiles in shoots and fibrous roots of *P. notoginseng* (Fig. S3). KEGG pathway enrichment analysis showed that increasing infection intensity led to significant down-regulation of growth-related DEGs, including those involved in carbon metabolism, in both shoots and fibrous roots (Fig. S4, Tables S5, and  S6). Conversely, some defense-related differentially expressed genes (DEGs), including those involved in jasmonic acid (JA) synthesis and plant–pathogen interactions, increased with infection intensity, indicating an activated defense response in shoots (Fig. 3A). It was also noted that most of these defense-related DEGs were significantly upregulated in fibrous roots after a two-leaf infection, but this upregulation was suppressed in fibrous roots with a three-leaf infection (Fig. 3A).

Quantitative hormone analysis showed that increasing the intensity of foliar infection led to a significant decrease in indole acetic acid (IAA) and abscisic acid (ABA) in shoots and fibrous roots, particularly IAA (*P* < 0.01) (Fig. 3B). A gradually increased content in JA and jasmonic acid-isoleucine (JA-Ile) was seen in both shoots and fibrous roots with one- or two-leaf infection, but JA and JA-Ile accumulated in high concentrations exclusively in shoots with three-leaf infection (Fig. 3B), suggesting foliar infection by *A. panax* with one- or two-leaf activates JA-mediated defense from shoots to roots.

### Foliar *A. panax* infection and exogenous JA modulated similar metabolic processes in the roots

**Figure 4:**
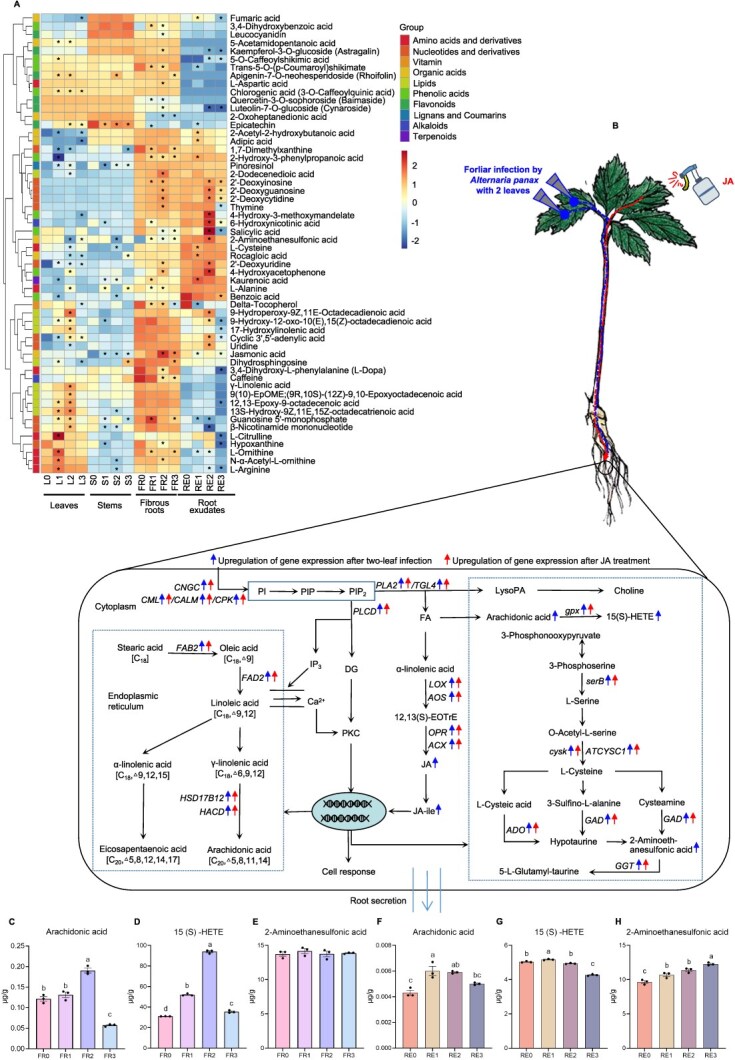
Effects of foliar infection by the weakly pathogenic strain AL with different intensities (inoculated zero-, one-, two-, or three leaf) on *P. notoginseng* metabolism. (A) Heatmap of metabolomic changes in leaves, stems, fibrous roots, and root exudates following foliar infection. Asterisks (*) indicate significant differences from control (*n* = 3, VIP ≥ 1, fold change ≥2 or ≤ 0.5). Sample identifiers: L0–L3 for leaves; S0–S3 for stems; FR0-FR3 for fibrous root; RE0-RE3 for root exudates, corresponding to the number of leaves infected (0, 1, 2, and 3). (B) Key gene expression in *P. notoginseng*’s fibrous roots induced by exogenous JA and *A. panax* infection (*n* = 3). (C–H) Metabolite profiles in JA-related pathways in fibrous roots (C–E) and root exudates (F–H) following infection by *A. panax* with different intensities. Data presented as mean ± SEM. Letters indicate significant differences (ANOVA with Tukey’s HSD, *P* < 0.05, *n* = 3).

Given the critical role of metabolism and root exudates in plant responses to *A. panax* infection and their influence on the rhizosphere microbiome, we conducted a comprehensive targeted metabolome analysis on samples from leaves, stems, fibrous roots, and root exudates after a 24-h infection with *A. panax* AL across varying intensities (zero-, one-, two-, or three leaf). PCA revealed distinct metabolic profile for leaves, stems, fibrous roots, and root exudates in infected plants compared to uninfected control (Fig. S5). Among the 56 differential metabolites identified, which correlated with enriched KEGG pathways from the transcriptome data, 14 differential metabolites, predominantly phenolic acids, and flavonoids, were more abundant in leaves, stems, and fibrous roots (Fig. 4A). Eleven metabolites, including lipids, amino acids, and nucleotides and derivatives, showed an increased abundance in leaves and fibrous roots. Additionally, 21 metabolites, mainly phenolic acids and nucleotides and their derivatives, were highly abundant in fibrous roots and root exudates (Fig. 4A). These results indicate that most of these metabolites were highly abundant in fibrous roots, suggesting that root is the primary metabolic site after leaf pathogen infection.

Due to the moderate infection intensity inducing enrichment of beneficial microbes, we further analyzed metabolites in the enriched pathways after a two-leaf infection by *A. panax*. By integrating transcriptome and metabolome data, we identified significant alterations in genes and metabolites involved in pathways such as alpha-linolenic acid metabolism in shoots and taurine and hypotaurine metabolism in fibrous roots (Fig. S6A and B). Notably, levels of 2-aminoethanesulfonic acid in taurine and hypotaurine metabolism increased significantly in fibrous roots and root exudates (Fig. 4A). Furthermore, metabolites related to alpha-linolenic acid metabolism, including JA, 2-dodecenedioic, arachidonic acid, 15(S)-hydroxy-5Z,8Z,11Z,13E-eicosatetraenoic acid (15(S)-HETE), 9-hydroxy-12-oxo-10(E),15(Z)-octadecadienoic acid, and cis-5,8,11,14,17-eicosapentaenoic acid, were also significantly upregulated in fibrous roots or root exudates (Figs 4A and S6C–F).

Targeted quantitative analysis confirmed significantly increased concentrations of key metabolites including 2-aminoethanesulfonic acid (FR: 13.7 ~ 14.2 ng/g; RE: 9.6 ~ 12.3 ng/g), arachidonic acid (FR: 0.05 ~ 0.13 ng/g; RE: 0.004 ~ 0.006 ng/g) and 15 (S) -HETE (FR: 31 ~ 94 ng/g; RE: 4.2 ~ 5.2 ng/g) in fibrous roots (FR) and root exudates (RE) after leaf pathogen infection (Fig. 4C–H). Interestingly, the expression of genes in the pathways associated with these key metabolites was significantly upregulated in fibrous roots following exogenous JA application to leaves (Figs 4B, S7, S8, S9, and  S10). JA application and foliar infection activated the same metabolic pathways in fibrous roots, highlighting JA's role in modifying root metabolism and exudation patterns postinfection.

### 2-aminoethanesulfonic acid responsible for enriching disease-suppressive rhizo-microbiota

**Figure 5:**
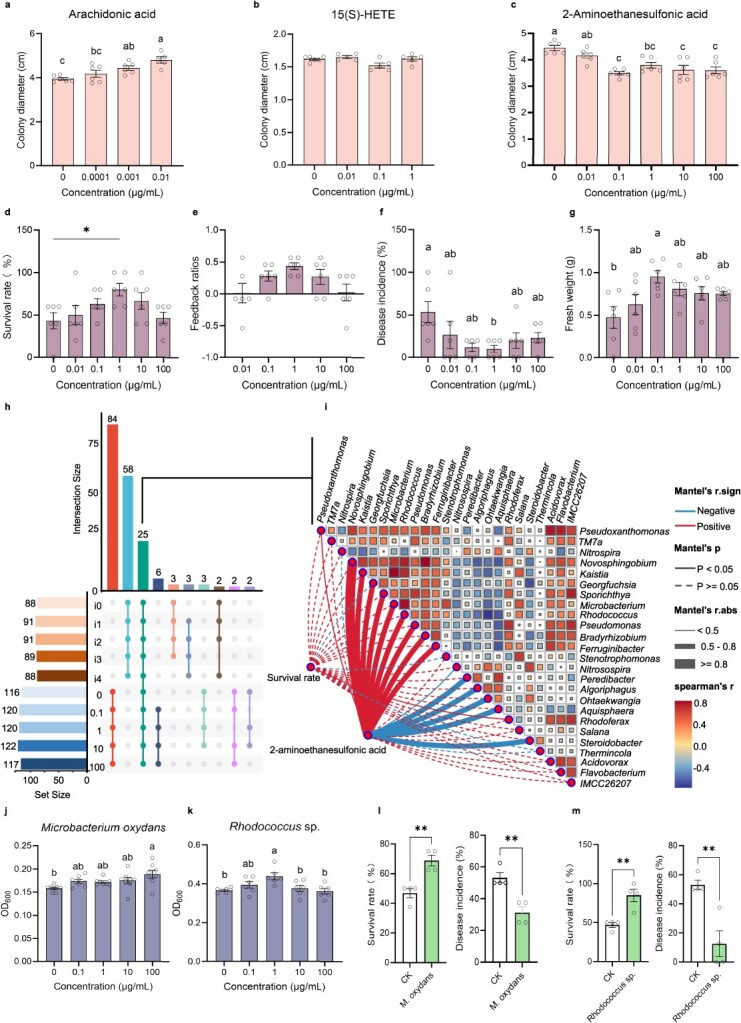
Effects of 2-aminoethanesulfonic acid in root exudates on the formation of disease-suppressive microbiota following foliar infection. (A–C) Effects of arachidonic acid (A) and 15 (S) -HETE (B), and 2-aminoethanesulfonic acid (C) on *Ilyonectria* sp.’s growth (*n* = 5, 6). (D–G) Effects of 2-aminoethanesulfonic acid on the survival rate (D), feedback ratio (E), disease incidence (F), and fresh weight (G) of *P. notoginseng* in the continuously cultivated soil (*n* = 6). (H–I) Differential bacteria genera co-influenced by foliar infection with *A. panax* and 2-aminoethanesulfonic acid amendment (0–100 μg/ml) (H) and their correlation with survival rate (I) (*n* = 6)*.* (J and K) Effects of 2-aminoethanesulfonic acid on growth of *M. oxydans* (J) and *Rhodococcus* sp. (K) (*n* = 8 and 6, respectively). (L and M) Effects of *M. oxydans* (L) and *Rhodococcus* sp. (M) on survival rate and root rot incidence of *P. notoginseng* (*n* = 6). CK denotes the survival ratio in pots with sterilized water. Letters indicate significant differences (ANOVA with Tukey’s HSD, *P* < 0.05); asterisks denote significant differences compared with blank control (two-tailed *t*-test, ^*^P < 0.05; ^**^P < 0.01).

The alterations of root exudates can significantly influence the rhizosphere microbiome [25]. Consequently, we first evaluated the effects of arachidonic acid, 15 (S)-HETE, and 2-aminoethanesulfonic acid within the rhizosphere concentration range on the growth of *Ilyonectria* sp. The results showed that arachidonic acid significantly enhanced the growth of *Ilyonectria* sp. (Fig. 5A). 15 (S)-HETE did not exert a significant influence on the growth of *Ilyonectria* sp. (Fig. 5B). 2-aminoethanesulfonic acid significantly inhibited the growth of *Ilyonectria* sp. (Fig. 5C). Relative abundance of *Ilyonectria* decreased following foliar infection by *A. panax* with weakly pathogenicity or a moderate level (Fig. 2B and C). Thus, we further analyzed the effects of 2-aminoethanesulfonic acid on the growth of *P. notoginseng* and the development of disease-suppressive rhizo-microbiota.

Application of 2-aminoethanesulfonic acid, especially at 1 μg/ml, significantly improved *P. notoginseng* survival, reduced root rot incidence, and increased biomass in soils that had been continuously cultivated with *P. notoginseng* (Fig. 5D-G). These findings suggest the presence of positive PSF. Furthermore, we analyzed the composition and function of microbial communities in the rhizosphere soil of *P. notoginseng* following soil amended with 2-aminoethanesulfonic acid at concentration from 0 to 100 μg/ml. We observed significant shifts in bacterial (*P* = 0.001) communities, as shown by PCoA of OTUs (Fig. S11). Twenty-five differential bacteria genera were found to be co-influenced by *A. panax* infection and 2-aminoethanesulfonic acid amendment (Fig. 5H). Among them, nine bacterial genera, including *Novosphingobium*, *Kaistia*, *Georgfuchsia*, *Sporichthya*, *Microbacterium*, *Rhodococcus*, *Pseudomonas*, *Bradyrhizobium*, and *Ferruginibacter*, exhibited a significant positive correlation (*r* > 0.5, *P* < 0.05) with the treatment concentration of 2-aminoethanesulfonic acid (Fig. 5I). This correlation also extended to the survival rates in the soil treated with 2-aminoethanesulfonic acid, with the exception of *Georgfuchsia* (Fig. 5I). Most of them, including *Microbacterium*, *Pseudomonas, Rhodococcus, Kaistia*, *Novosphingobium*, and *Bradyrhizobium,* were enriched by *A. panax* with a moderate level of infection or weakly pathogenicity (Fig. 2D). *Rhodococcus* sp. and *Microbacterium oxydans* together enhanced the antagonistic response against *Ilyonectria* sp. (Fig. 2G). *In vitro* experiments demonstrated that 2-aminoethanesulfonic acid significantly promoted the growth of the core beneficial microbes *M. oxydans* in a concentration-dependent manner, as well as *Rhodococcus* sp. within the concentration range present in the rhizosphere (Fig. 5J and K). These species significantly improved *P. notoginseng* survival and reduced root rot incidence when introduced to the rhizosphere (Fig. 5L and M). These results indicate that 2-aminoethanesulfonic acid significantly improved the survival rate and biomass of *P. notoginseng* by modulating the rhizosphere microbial community, reducing the incidence of root rot, and thereby playing a crucial role in maintaining plant health and growth.

## Discussion

Agroforestry represents a typical system with a high biodiversity. In this system, the diverse interactions among plants, animals and microorganisms contribute to stability and plant health. Here, we found that a moderate level of infection with *A. panax* or exposure to weakly pathogenicity isolates induced the recruitment of a disease-suppressive rhizo-microbiota, thereby enhancing plant health and promoting positive PSF. The activation of JA-mediated metabolomic pathways, which is a response to foliar pathogen infection extending from shoots to roots, includes the synthesis and secretion of 2-aminoethanesulfonic acid. This process induces the rhizosphere for the assembly of a disease-suppressive rhizo-microbiota, contributing to positive PSF. However, excessive foliar pathogen infection or exposure to high pathogenicity isolates confined the JA-mediated defense response to the shoots, disrupting this rhizomicrobial enhancement and leading to negative PSF. This study elucidates the ecological role of foliar pathogen infestation in regulating PSFs within agroforestry systems. It also suggests a potentially effective strategy for enhancing soil health in agricultural production: using foliar inducers associated with JA to trigger the assembly of disease-suppressive microbiota in the rhizosphere.

Although the link between plant diversity and above-ground plant productivity has been extensively studied, there is a growing recognition that many mechanisms underlying positive biodiversity-ecosystem functioning relationships occur below-ground [26, 27]. Consequently, the significance of plant–soil feedback loops is becoming increasingly recognized [2]. Our field and greenhouse experiments revealed that foliar infection by a weakly pathogenic strain of *A. panax* or at a moderate infection intensity led to the development of disease-suppressive soil, resulting in positive PSF. Conversely, a highly pathogenic strain of *A. panax* or excessive infection intensity exhibited negative PSF. These findings highlight that the pathogenicity and infection intensify diversity of *A. panax* significantly affects PSF in agroforestry systems, thereby enhancing our comprehension of how plant–microbial diversity interactions contribute to stability. Increasing evidence suggests plant diversity influences the belowground microbial communities that consecutively promote ecosystem functioning both below- and above-ground [28, 29]. A series of studies have demonstrated that plants exposed to pathogen infection can attract antagonistic microbial populations by the 'cry for help' strategy, leading to the formation of a disease-suppressive environment, thereby reducing the severity of disease [30]. In this study, foliar infection by weakly pathogenicity isolate of *A. Panax* AL elicited the enrichment of beneficial bacteria, such as *Microbacterium*, *Mycobacterium Pseudoxanthomonas, Pseudomonas, Rhodococcus, Allocatelliglobosispora, Flavobacterium,* and *Nocardioides.* These beneficial bacteria have been partially endorsed by other studies [31–34]. We have found these taxa not only suppressed *Ilyonectria* pathogen but also enhanced the survival of *P. notoginseng*. These results underscore the significant role of soil microorganisms in mediating the effects of plant–foliar pathogen diversity interactions on PSF within agroforestry systems.

Numerous studies have shown that disease suppression is typically the result of the collective activity of a microbial microbiota rather than the action of a single bacterial or fungal species [35, 36]. In this study, the combined effect of *Rhodococcus* sp. and *Microbacterium oxydans* demonstrated a significantly enhanced antagonistic response against *Ilyonectria* sp. Similarly, simplified synthetic microbiota derived from keystone species in the *P. notoginseng* rhizosphere exhibited higher antagonistic activity against the colony growth of *I. destructans* compared to individual isolates [37]. These findings demonstrated the synergistic potential of the core rhizosphere microbiota in suppressing soil-borne pathogens. Diverse microbial taxa play crucial roles in protecting host plants from disease infections through various mechanisms, including the production of antimicrobial components, competition for nutrients and ecological niches, biofilm formation, and activation of plant defense responses [38, 39]. While this study identifies functional roles and co-occurrence patterns of core taxa such as *Rhodococcus* sp. and *M. oxydans*, their precise protective mechanisms and functional contributions to pathogen suppression necessitate further experimental validation.

Plants communicate with rhizosphere microbes through root exudates, which are influenced by pathogen infections and immune responses [40, 41]. Our study demonstrates that moderate foliar infection activates JA-mediated root metabolism and exudate secretion, which facilitates the recruitment of disease-suppressive microbiota in the rhizosphere. However, the molecular mechanisms governing the long-distance ‘leaf-to-root’ signaling remain to be further explored. Recent research suggests that jasmonates may serve as systemic signaling molecules transmitted along the phloem, with their intercellular movement mediated by AtJAT3/AtJAT4 homo−/heterodimers [42]. Grafting experiments and hormone profiling revealed that 12-oxophytodienoic acid (OPDA), a bioactive precursor of JA, translocates from wounded shoots to roots through the phloem, a process dependent on JASSY, a chloroplast export protein critical for OPDA transport [43, 44]. Core JA signaling components, including transcription factor MYC2 and JA-ZIM-domain (JAZ) [45, 46], likely regulate the synthesis and secretion of root metabolites (e.g. tanshinone, phenolic acid, and 2-aminoethanesulfonic acid), thereby creating rhizosphere chemical ‘hotspots’ to enrich beneficial microbes. This process may involve direct or indirect interaction between JA signaling genes and promoters of metabolic enzyme genes such as those involved in organic acid synthesis in root cells [22, 47]. However, the precise regulatory network remains to be further elucidated.

Furthermore, this study reveals consistency in metabolic pathways activation triggered by both foliar infection and exogenous JA application, highlighting the promise of JA analogs as eco-friendly elicitors for enhancing beneficial microbiota assembly and inhibiting root rot pathogen, contributing to the sustainable management of soilborne diseases. For instance, methyl jasmonate (MeJA) treatment in broccoli increased root secretion of glucosinolates and isothiocyanates, thereby inhibiting the growth of the pathogen *Fusarium oxysporum* [48]. Moreover, MeJA promoted biofilm formation in soil microbial communities, which promotes host plant growth [49]. Building on these insights, future research could integrate synthetic biology-driven approaches to design plant–microbe co-response modules, such as designing JA-responsive plant lines that secrete pathogen-antagonistic metabolites upon immune activation or modifying rhizosphere microbiota to rewiring rhizosphere microbiomes with engineered strains expressing JA signal-responsive biosynthetic pathways. These directions will drive the transition of ‘plant–microbe collaborative disease resistance’ from mechanistic research to smart field control, providing innovative solutions for sustainable agriculture.

In addition to JA, salicylic acid (SA) also plays a pivotal role in immune responses and in shaping rhizosphere microbial composition [50]. Although JA and SA signaling pathways are traditionally viewed as antagonistic [51], emerging evidence suggests their synergistic effects in coordinating plant immunity responses [52]. A striking example is observed in rice (*Oryza sativa*) infected by *M. oryzae* and *Xanthomonas oryzae* pv. *oryzae,* where both SA and JA levels are concurrently elevated [51]. This combined action of SA and JA, mediated through transcriptional regulators OsNPR1 and OsMYC2/3, synergistically enhances rice resistance to viruses [53]. Crucially, SA- and JA-driven immune responses can alter root exudate composition, thereby reshaping rhizosphere microbial community structure [54, 55]. Thus, this crosstalk not only enhances plant resistance to pathogens but may also improve plant fitness through microbiome-mediated benefits. These findings emphasize the need to explore how SA–JA crosstalk coordinates immune outputs and microbiome assembly, potentially unlocking hormone-guided strategies to engineer disease-suppressive microbiomes for crop protection.

The strategic development of disease-suppressive soil involves a delicate balance between plant growth and defense mechanisms [56]. We found that as infection intensity increases, gene expression related to plant growth and IAA production decreased, while defense-related genes and hormones like JA and JA-Ile increased. This shift towards defense at the expense of growth is part of the plant's adaptive response to stress [57]. Notably, the leaf-root linkage in JA-mediated defense is activated with one- or two-leaf infected, enhancing recruitment of disease-suppressive microbiota. However, with four-leaf infected, the defense response in roots is reduced, weakening the recruitment of beneficial microorganisms. These results suggest that plants navigate a complex trade-off in allocating resources from leaf to root for growth and defense. Yet, the mechanisms regulating the growth-defense balance from leaf to root are not fully understood. A previous study demonstrated that JA-mediated MYB transcription factor 1 (JMTF1) mediates the interplay between JA and auxin signaling in rice defense response [58]. In this study, we observed significant upregulation in the expression of JA-related transcription factors, including the pathogenesis-related genes transcriptional activator (PTI6) [59], and the WRKY transcription factor (WRKY) [60], following foliar infection by *A. panax*. Their roles in balancing plant growth and defense, especially in recruiting beneficial microorganisms, require further investigation.

Prebiotics, which enhance beneficial gut microbes, can also bolster plant stress resistance by modulating rhizosphere microbiomes [61, 62]. Foliar infections increase secretion of organic acids, sugars, and amino acids to regulate rhizosphere microbiome in a previous study [5]. This study reveals that 2-aminoethanesulfonic acid, induced by exogenous JA and foliar infection, inhibits *Ilyonectria* sp. growth and enriches disease-suppressive microbiota, such as *Rhodococcus* sp. and *Microbacterium oxydans*. In *P. notogening*, low concentrations of 2-aminoethanesulfonic acid significantly improve seedling survival and biomass in continuously cultivated soil, suggesting a mechanism for recruiting disease-suppressive microbiota, developing positive PSF after foliar infection [63]. Additionally, arachidonic acid, a JA-mediated product, impacts soilborne pathogen growth and may act as an elicitor for plant defense [64]. Further research is required to understand its role in JA-mediated exudate–microbiome interactions. These findings highlight the potential of using 2-aminoethanesulfonic acid and arachidonic acid as prebiotics and elicitors to trigger plant defense mechanisms and recruit beneficial microbiota for robust seedlings growth and sustainable soilborne disease management.

## Conclusions

**Figure 6:**
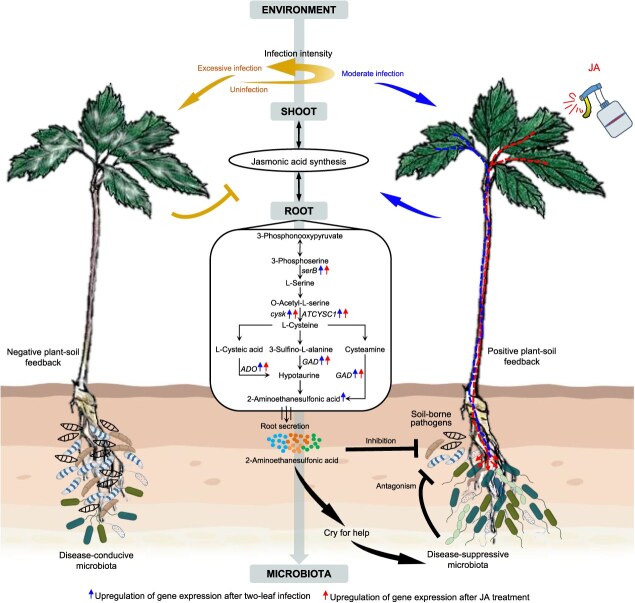
Mechanistic model of moderate foliar infection by *A. panax,* recruiting beneficial rhizosphere microorganisms to alleviate negative plant–soil feedback along the environment-leaf-root-microbiome axis.

Our study demonstrates that foliar pathogens with weakly pathogenicity or moderate level of infection trigger a ‘cry for help’ strategy in plants, recruiting disease-suppressive rhizo-microbiota to balance growth and defense mechanisms. This strategy, facilitated by JA-induced metabolic responses that act systemically from shoots to roots, influences PSF in agroforestry systems. However, excessive foliar pathogen infection disrupts the beneficial effects of rhizomicrobial activity (Fig. 6). Our findings reveal the ecological roles of foliar pathogens in affecting PSF and suggest that JA-pathway triggers could serve as potent alternatives to virulent pathogens, enabling the assembly of disease-suppressive microbiota. This approach contributes to the ecological prevention and control of soilborne diseases, enhancing the productivity and sustainability of agricultural production systems.

## Materials and methods

### Evaluation of plant–soil feedback in plots with different levels of Alternaria leaf spot in agroforestry system

To clarify the effects of leaf spot with different levels on plant–soil feedback (PSF) in agroforestry system, three-year-old *P. notoginseng* exhibiting Alternaria leaf spot was collected from the Organic Sanqi Planting Base located beneath *Pinus yunnanensis* forests in Xundian County, Yunnan, China (103.13°E, 25.67°N; altitude of 1960 m). The severity of the incidence is categorized into mild (partial defoliation) and severe (complete leaf loss) leaf spot (Fig. 1A). Healthy *P. notoginseng* plants without any disease symptoms served as control (Fig. 1A). Each treatment contained ten *P. notoginseng*. The infection*-*conditioned rhizosphere soils of *P. notoginseng* from agroforestry systems were used to assess PSF by analyzing the survival rate and dry biomass of subsequent *P. notoginseng* (see description in Appendix S1).

### Isolation of different pathogenicity *A. panax* in agroforestry system and its effects on PSF and rhizosphere microbiome

To determine the pathogenic diversity of leaf spot pathogen *A. panax* in agroforestry system, *A. panax* was isolated from the leaves of *P. notoginseng* with leaf spot symptoms. The isolation method is described in detail in a previous study [65]. The pathogenicity of *A. panax* isolates was determined on the leaves of *P. notoginseng in vitro* and *in vivo* (see description in Appendix S2).

To clarify the effects of pathogenic diversity of *A. panax* on PSF, three strains of *A. panax* (A5, A3, and A4), which specifically represent low (AL), medium (AM), and high (AH) levels of pathogenicity, according to pathogenicity determination, were selected for further study. An *A. panax* (AH, AM, or AL) mycelial block (6 mm diameter) was placed face down onto the pre-made wound on leaves of six-month-old *P. notoginseng* seedlings, and two leaves of each seedling were inoculated (Fig. 1G, Step 1). Healthy plants were used as control. Each treatment consisted of 24 pots (10.0 × 12.5 × 14.5 cm), with eight seedlings planted per pot. All pots were prewrapped in clear plastic bags to prevent contamination of the soil by *A. panax* and were placed in transparent boxes and incubated with a photoperiod of 16 h light/8 h dark at 25 ± 2°C. When the leaves showed symptomatic lesions, the rhizosphere soil was sampled following a previously described procedure [37]. Each treatment had six biological replicates, and each replicate contained four pots. All treatments were collected and stored at −80°C for microbial analysis (see description in Appendix S3). The remaining soil, defined as bulk soil, was used to evaluate the feedback relationship between *P. notoginseng* and the infection*-*conditioned soil according to previous methods [1]. Briefly, the bulk soil from a treatment was divided into two parts. One was steamed at 90°C for 2 h, while the other remained untreated. Subsequently, the soil was placed separately in pots (10.0 × 12.5 × 14.5 cm). Healthy seedlings were transplanted into the bulk soil and the seedling survival rate was recorded. Feedback ratios were calculated for each replicate pair as (Ai-Bi)/maximum (Ai, Bi), where A and B are survival rates obtained in soil treatments with or without sterilization (Fig. 1G).

### Effects of different intensities of *A. panax* infection on PSF and rhizosphere microbiome

To eliminate variables associated with strains with different levels of pathogenicity, we employed the weakly pathogenic strain AL for studying the effects of different infection intensity on PSF. Leaves of six-month-old *P. notoginseng* seedlings were inoculated with 0, 1, 2, 3, or 4 mycelial blocks of strain AL in the manner mentioned above (Fig. 1G, Step 2). Each treatment consisted of 24 pots (10.0 × 12.5 × 14.5 cm), with eight seedlings planted per pot. All pots proceeded in the same manner as previously mentioned. When the leaves showed symptomatic lesions, the rhizosphere soil was sampled following a previously described procedure [37]. Each treatment had six biological replicates, and each replicate contained four pots. All treatments were collected and stored at −80°C for microbial analysis (see description in Appendix S3). The functions of the microorganisms modified by foliar infection by *A. panax* were evaluated based on the results of the microbial analysis (see description in Appendix S4). The remaining soil was also used to evaluate the feedback relationship between *P. notoginseng* and the infection*-*conditioned soil according to previous methods [1].

### Effects of different intensities of *A. panax* infection on metabolic and transcriptome profiles

To analyze how plants regulate rhizosphere microbes responsible for PSF, metabolism and transcriptome of *P. notoginseng* were analyzed after different intensities of leaf infection by *A. panax* AL. Eight clean healthy seedlings were transferred into 60 ml sterile distilled water in a glass pot wrapped in tinfoil. Each seedling, 0, 1, 2, and 3 leaves were inoculated in the manner mentioned above. Each treatment consisted of three biological replicates, with one replicate consisting of 12 pots (10.0 × 12.5 × 14.5 cm). All pots were placed in transparent boxes and incubated with a photoperiod of 16 h light/8 h dark at 25 ± 2°C. After 24 h, the samples from leaves and fibrous roots were stored at −80°C for transcriptome analysis (see description in Appendix S5). Meanwhile, filter paper and 0.22-μm hydrophilic membranes were used to filter the solutions from the pots, and the dried materials were subsequently concentrated under low pressure as root exudates and stored at −80°C [66]. The samples from leaves, stems, fibrous roots, and root exudates were used for UPLC-MS/MS analyses (see description in Appendix S6). Differentially expressed genes (DEGs) and metabolites (DAMs) were identified, followed by principal component analysis (PCA) and mapping to KEGG pathways. Based on the findings from the transcriptome and metabolic analysis, we investigated alterations of hormone levels in leaves and roots (see description in Appendix S7). The expression of DEGs in fibrous roots was analyzed by quantitative real-time PCR (qRT-PCR) (see description in Appendix S8). The primers used were listed in Table S7. The content of three significantly changed metabolites (arachidonic acid, 15(S)-hydroxy-5z,8Z,11Z, 13e-eicosatetraenoic acid (15(S)-HETE), and 2-aminoethanesulfonic acid) in fibrous roots and root exudates was quantified using standard curves that showed the linear relationships between the peak areas and the concentrations (Table S8) (see description in Appendix S9).

### Effects of root secreted metabolites on the differential microorganisms and rhizosphere microbiome

We evaluated the growth of *Ilyonectria* sp. on PDA medium amended with arachidonic acid, 15(S)-HETE, or 2-aminoethanesulfonic acid (see description in Appendix S10). To confirm that 2-aminoethanesulfonic acid plays a crucial role in triggering the formation of disease-suppressive microbiota following foliar infection, a pot experiment was performed to assess the impacts of 2-aminoethanesulfonic acid on alleviating negative PSF and rhizosphere microbiome (see description in Appendix S11). To verify the function of 2-aminoethanesulfonic acid on enrichment of disease-suppressive microbes, we evaluated the effects of 2-aminoethanesulfonic acid on the growth of the core beneficial microbes *Microbacterium oxydans* and *Rhodococcus* sp. (see description in Appendix S12). To verify the beneficial effects of the core microbes, a greenhouse experiment was used to evaluate the growth of *P. notoginseng* after exogenous irrigating *Microbacterium oxydans* and *Rhodococcus* sp. (see description in Appendix S13).

### Statistical analysis

Data were analyzed using SPSS software version 17.0 (SPSS Inc., Chicago, IL, USA) and GraphPad Prism software 10 (GraphPad Inc., USA). Group differences were analyzed by two-sided ANOVA with Tukey’s multiple comparison test analysis, the Kruskal–Wallis test, or a two-tailed Student’s *t*-test, as appropriate. Correlations between survival rates of *P. notoginseng* and the microbes in rhizosphere soil were performed using Spearman correlation analysis.

## Supplementary Material

Web_Material_uhaf137

## Data Availability

All sequences of ITS and 16S rRNA genes of rhizosphere microbiome following different pathogenicity levels or intensity of foliar infection by *A. panax* can be found in the NCBI database under accession number PRJNA1104181. All sequences of ITS and 16S rRNA genes of rhizosphere microbiome following soil drenching with 2-aminoethanesulfonic acid at concentration of 0 to 100 μg/ml can be found in the NCBI database under accession number PRJNA1106096. All raw reads generated by transcriptome sequencing were deposited in the NCBI database with accession number PRJNA1104498. All data are available in the main text or the supplementary materials.
