# Supplementary material for: Diversified alternaria pathogenicity alters plant–soil feedbacks through leaf–root-microbiome dynamics in agroforestry systems
Source: Hortic Res. 2025 May 21;12(8):uhaf137. doi: 10.1093/hr/uhaf137 (PMC12282122; doi:10.1093/hr/uhaf137)
Supplement: Web_Material_uhaf137 [file web_material_uhaf137.zip › Supplementary figure.docx]

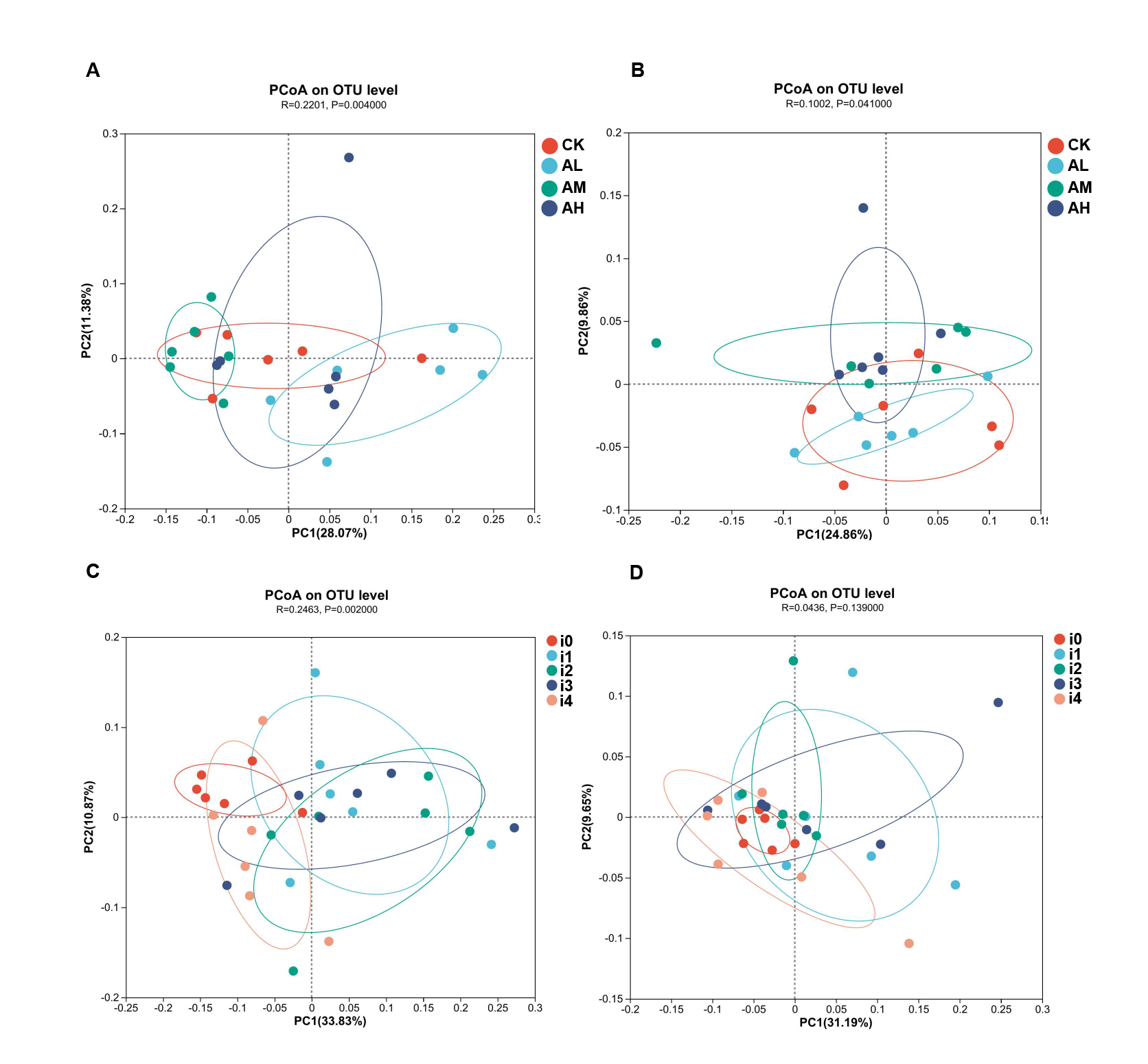


**Figure S1** PCoA analysisof the rhizosphere soil microorganisms following different pathogenicity levels (low, medium, and high) (A-B) and infection intensities (inoculated 0-4 leaves) (C-D) of *Alternaria panax* infecting *Panax notoginseng* leaves. AH, AM, and AL denote the pathogenicity levels of *A. panax*. i0, i1, i2, and i3 denote the number of leaves infected by *A. panax* AL. CK denotes blank control with healthy plants.


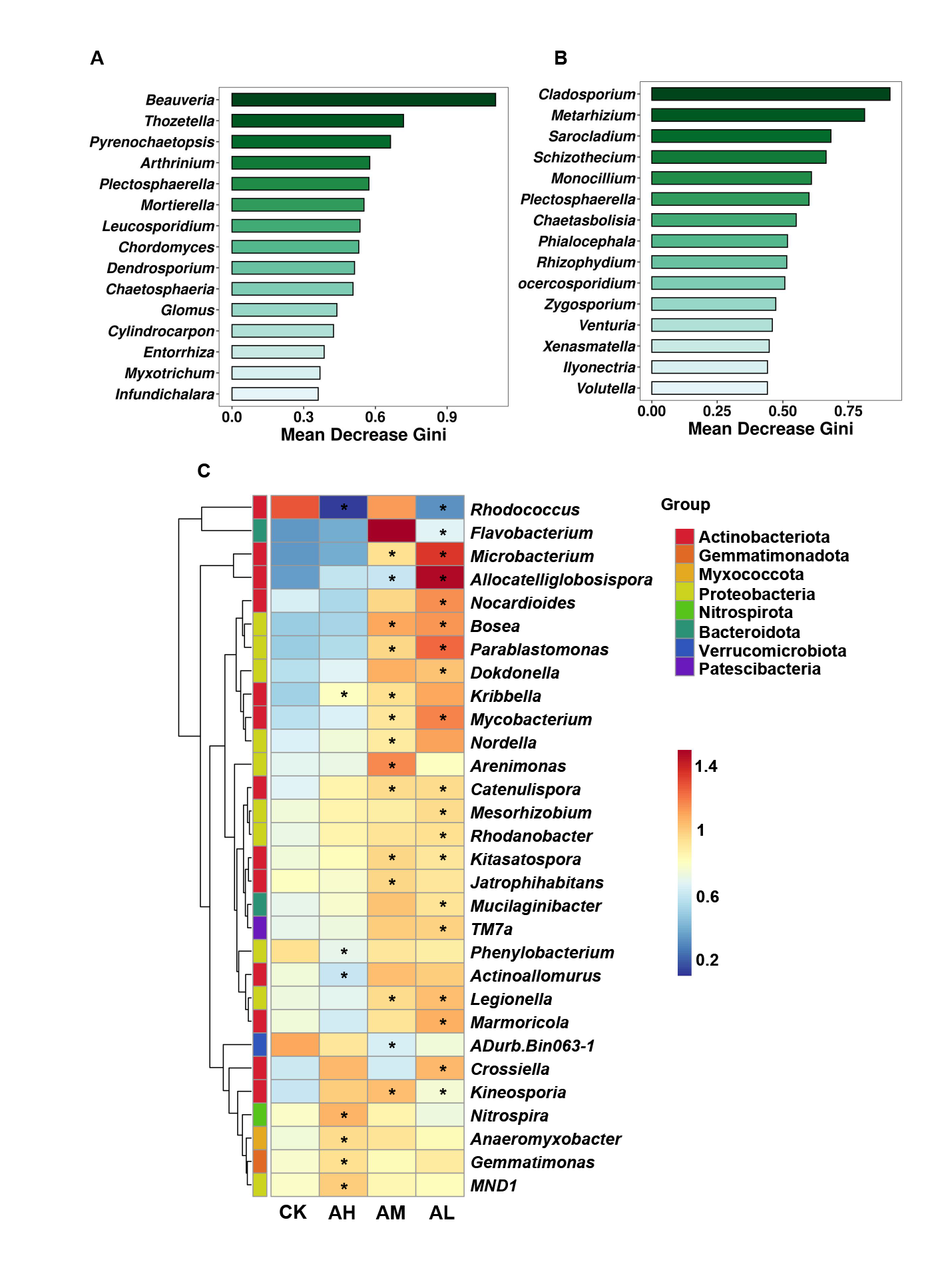


Figure S2 Differential genera in different pathogenicity levels (low, medium, and high) and infection intensities (inoculaed 0-4 leaves) of *Alternaria panax* infecting *P. notoginseng* leaves. (A-B) The top 15 biomarkers following infection by *A. panax* with different pathogenicity levels (A) and the varying infection intensities of AL (B) according to Random forest analysis. (C) Relative abundance shifts of the top 30 genera from differential bacteria after foliar infection by *A. panax* with different pathogenicity levels. Asterisks denote significant differences compared with control (two-tailed *t*-test, **P*<0.05; n = 6).


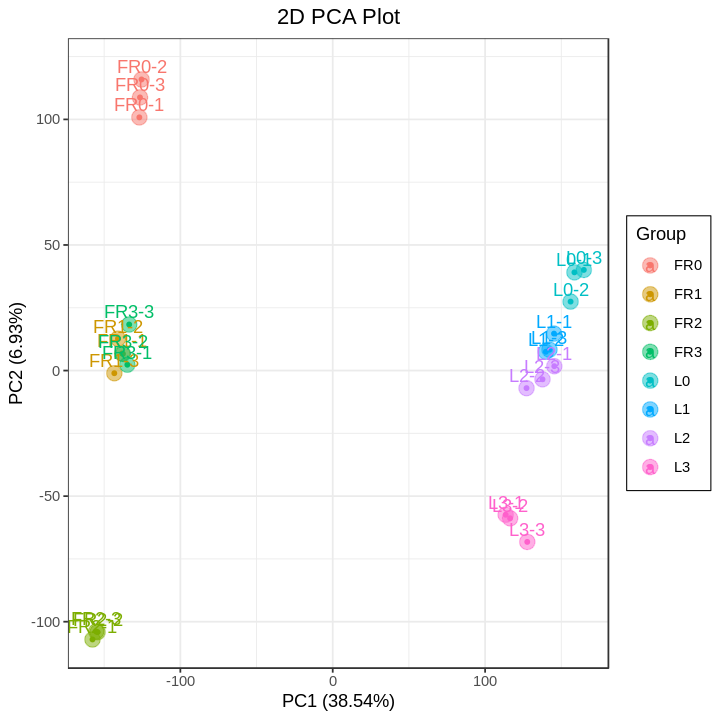


Figure S3 Principal component analysis (PCA) in gene expression profiles following foliar infection by *Alternaria panax* AL, exhibiting low level of pathogenicity, with different infection intensities, as presented as 0 (L0), 1 (L1), 2 (L2), and 3 (L3) leaves inoculated. FR0, FR1, FR2, and FR3 respectively represent the fibrous root samples L0 to L3 treatments.


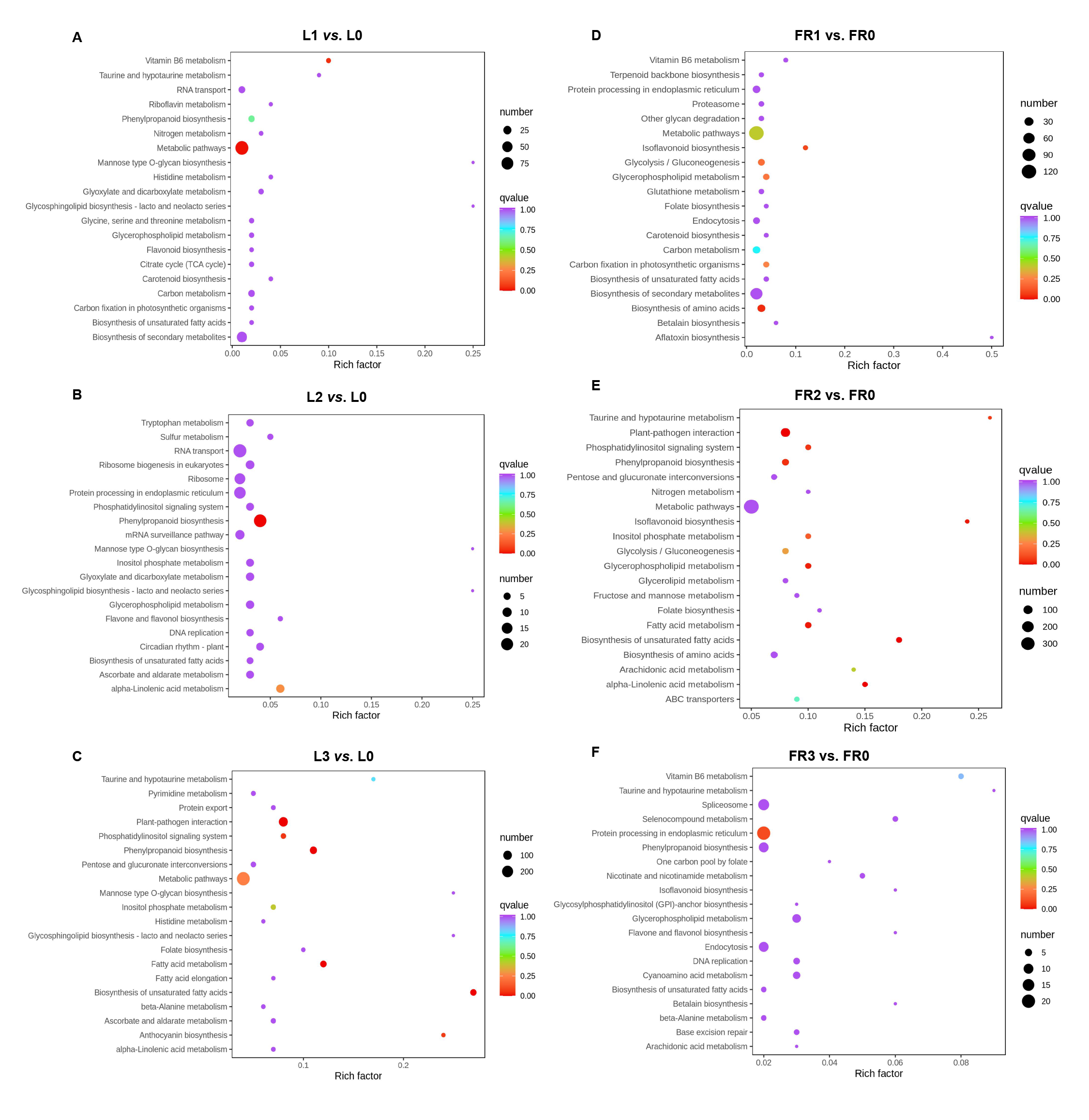


**Figure S4** Bubble diagram of differential gene enrichment in leaves (A-C) and fibrous roots (D-F) of notogensing following infection by *Alternaria panax* AL, exhibiting low level of pathogenicity, with different infection intensities, as presented as 0 (L0), 1 (L1), 2 (L2), and 3 (L3) leaves inoculated. FR0, FR1, FR2, and FR3 respectively represent the fibrous root samples L0 to L4 treatments.


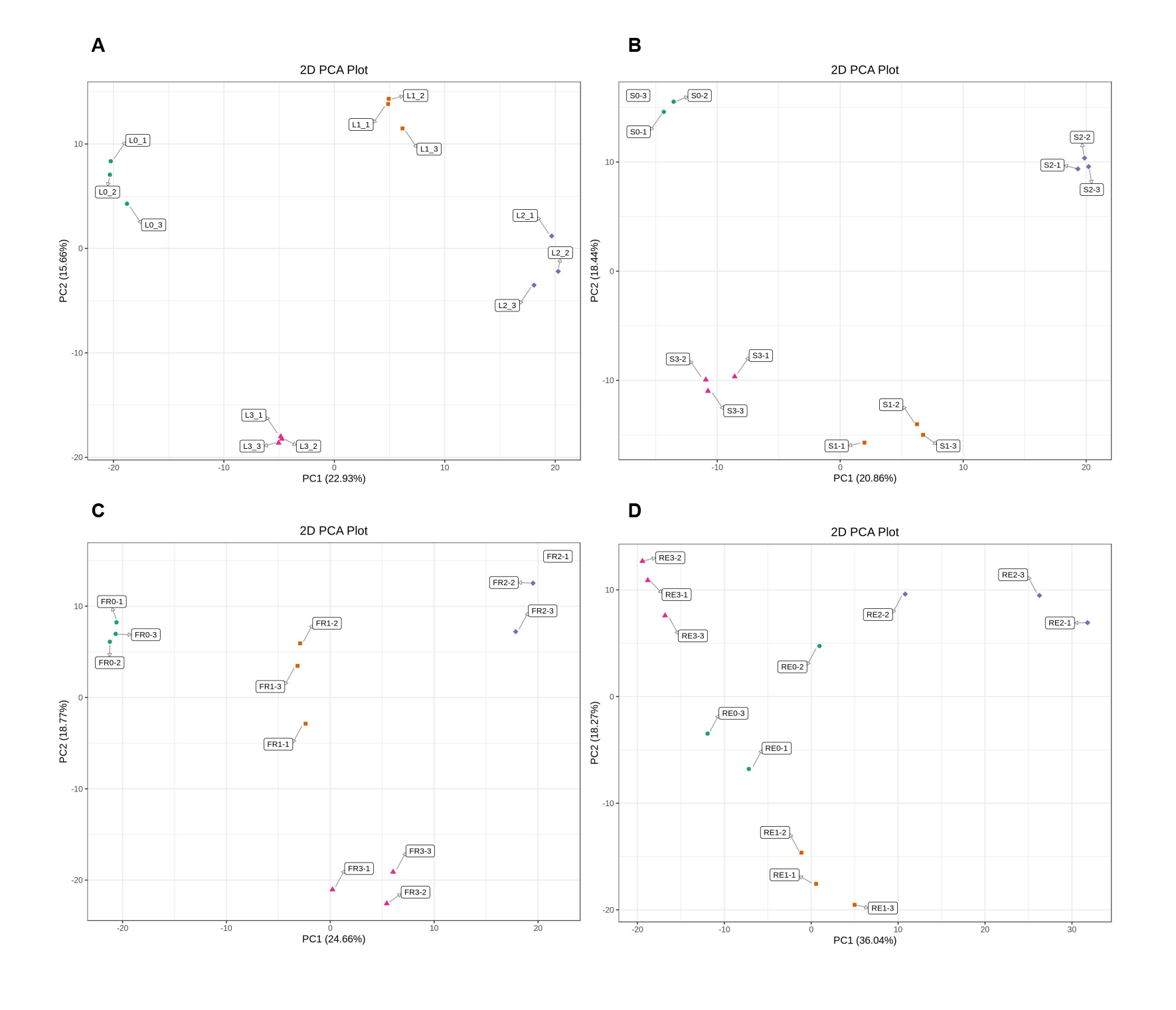


Figure S5 Principal component analysis (PCA) of the metabolic profiles in leaves (A), stems (B), fibrous roots (C) and root exudates (D) following foliar infection by *Alternaria panax* AL, exhibiting low level of pathogenicity, with different infection intensities. Sample identifiers: L0, L1, L2, and L3 for leaves; S0, S1, S2, and S3 for stems; FR0, FR1, FR2, and FR3 for fibrous root; RE0, RE1, RE2, and RE3 for root exudates, corresponding to the number of leaves infected (0, 1, 2, and 3).


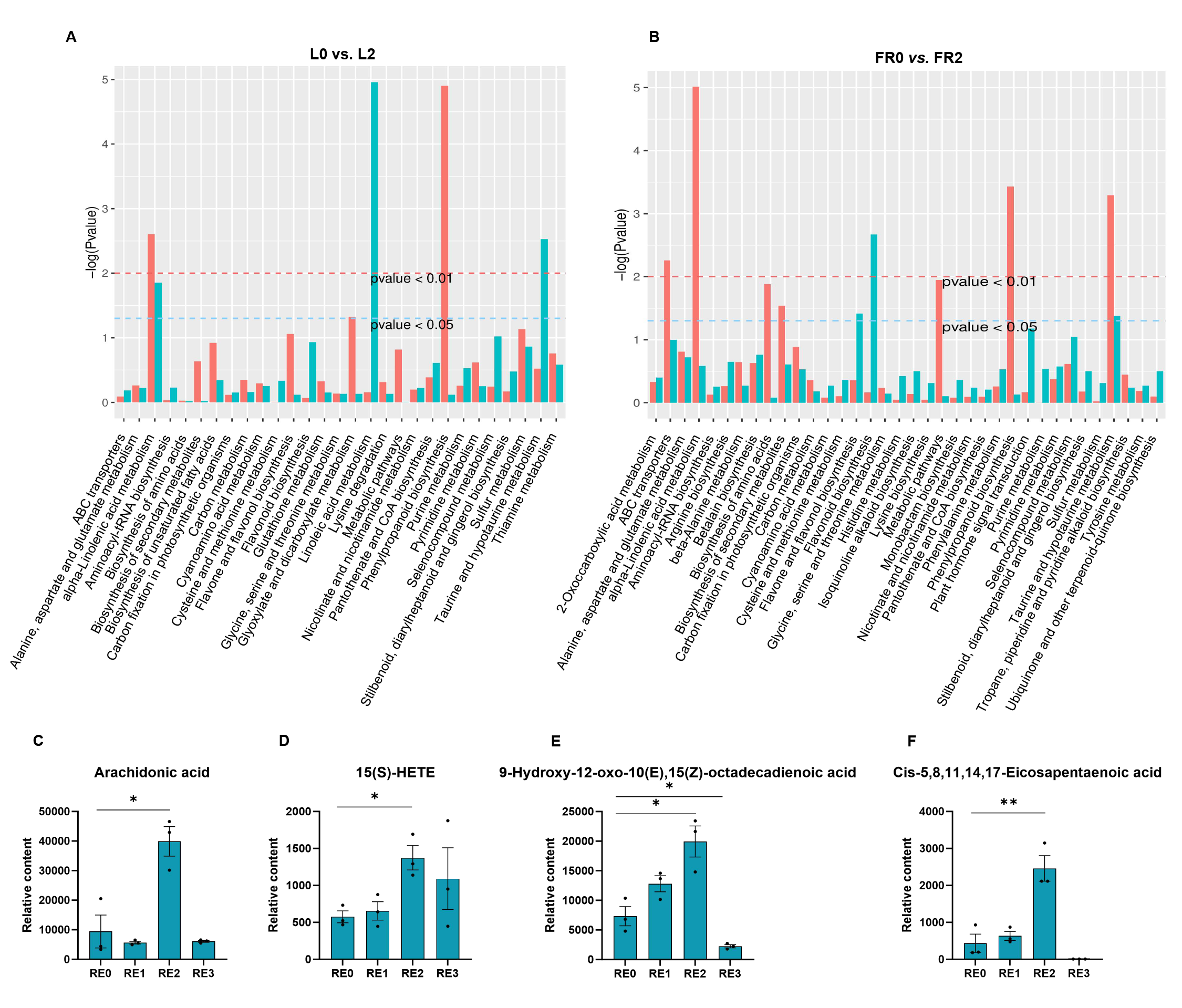


Figure S6 KEGG combined enrichment analysis in leaves (A) and fibrous roots (B) and related metabolites changes (C-F) in root exudates following foliar infection by *Alternaria panax* AL, with different infection intensities, as presented as 0 (L0), 1 (L1), 2 (L2), and 3 (L3) leaves inoculated. FR0, FR1, FR2, and FR3 respectively represent the fibrous root samples L0 to L3 treatments. RE0, RE1, RE2, and RE3 respectively represent the root exudates samples L0 to L3 treatments. The data are expressed as the mean ± SEM. Asterisks denote significant differences compared with control (two-tailed *t*-test, **P*<0.05; ***P*<0.01; n = 3).


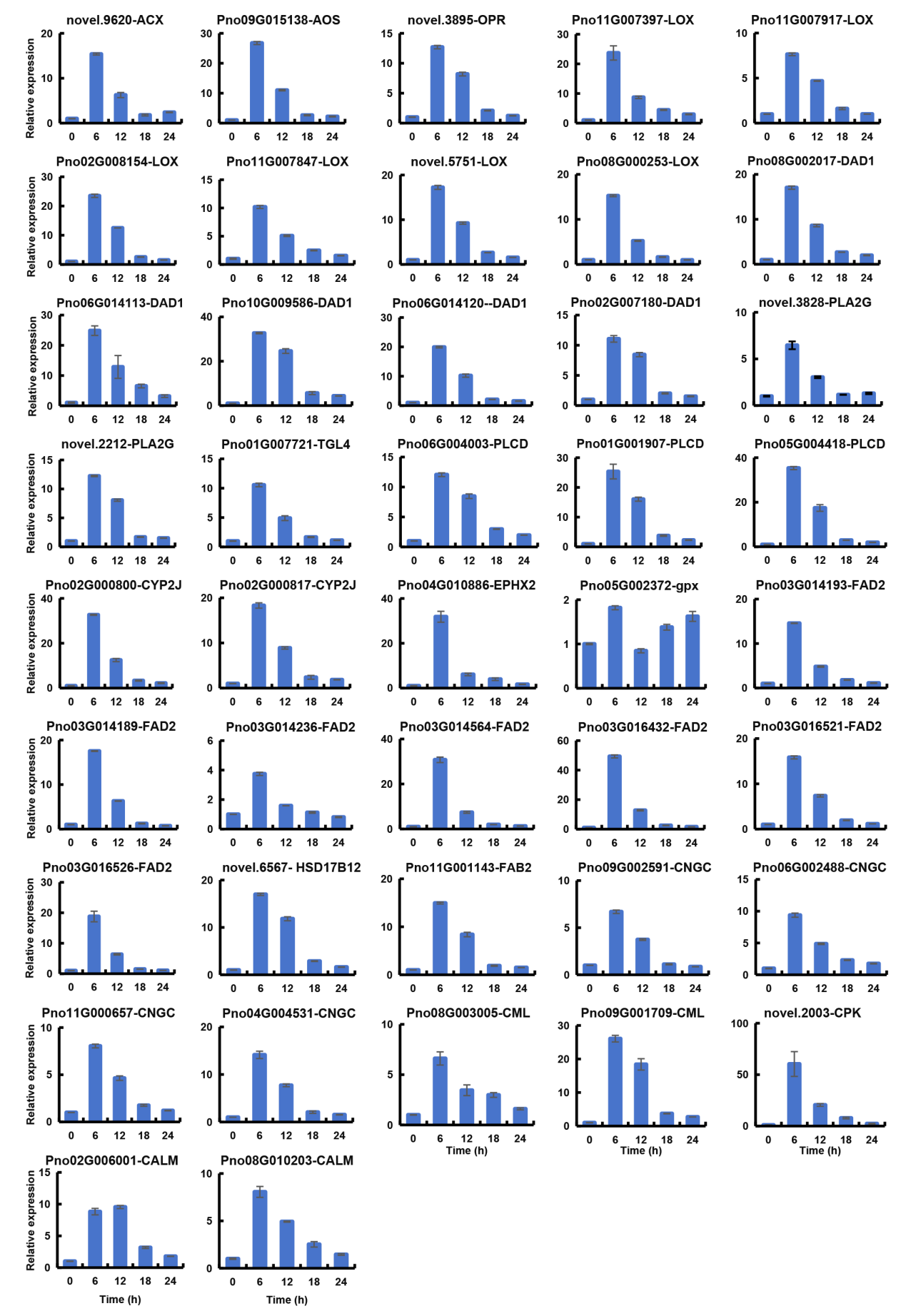


Figure S7 Key gene expression in *P. notoginseng*’s fibrous roots activated by foliar *Alternaria panax* infection, as determined by qRT-PCR analysis. The data are expressed as the mean ± SEM (n = 3). ACX: acyl-CoA oxidase; AOS: hydroperoxide dehydratase; OPR: 12-oxophytodienoic acid reductase; LOX: lipoxygenase; DAD1: phospholipase A1; PLA2G: secretory phospholipase A2; TGL4: TAG lipase / steryl ester hydrolase / phospholipase A2 / LPA acyltransferase; PLCD: phosphatidylinositol phospholipase C; CYP2J: cytochrome P450 family 2 subfamily J; EPHX2: soluble epoxide hydrolase / lipid-phosphate phosphatase; gpx: glutathione peroxidase; FAD2: omega-6 fatty acid desaturase / acyl-lipid omega-6 desaturase (Delta-12 desaturase); HSD17B12: 17 beta-estradiol 17-dehydrogenase / very-long-chain 3-oxoacyl-CoA reductase; FAB2: acyl-[acyl-carrier-protein] desaturase; CNGC: cyclic nucleotide gated channel; CML: calcium-binding protein; CPK: calcium-dependent protein kinase; CALM: calmodulin.


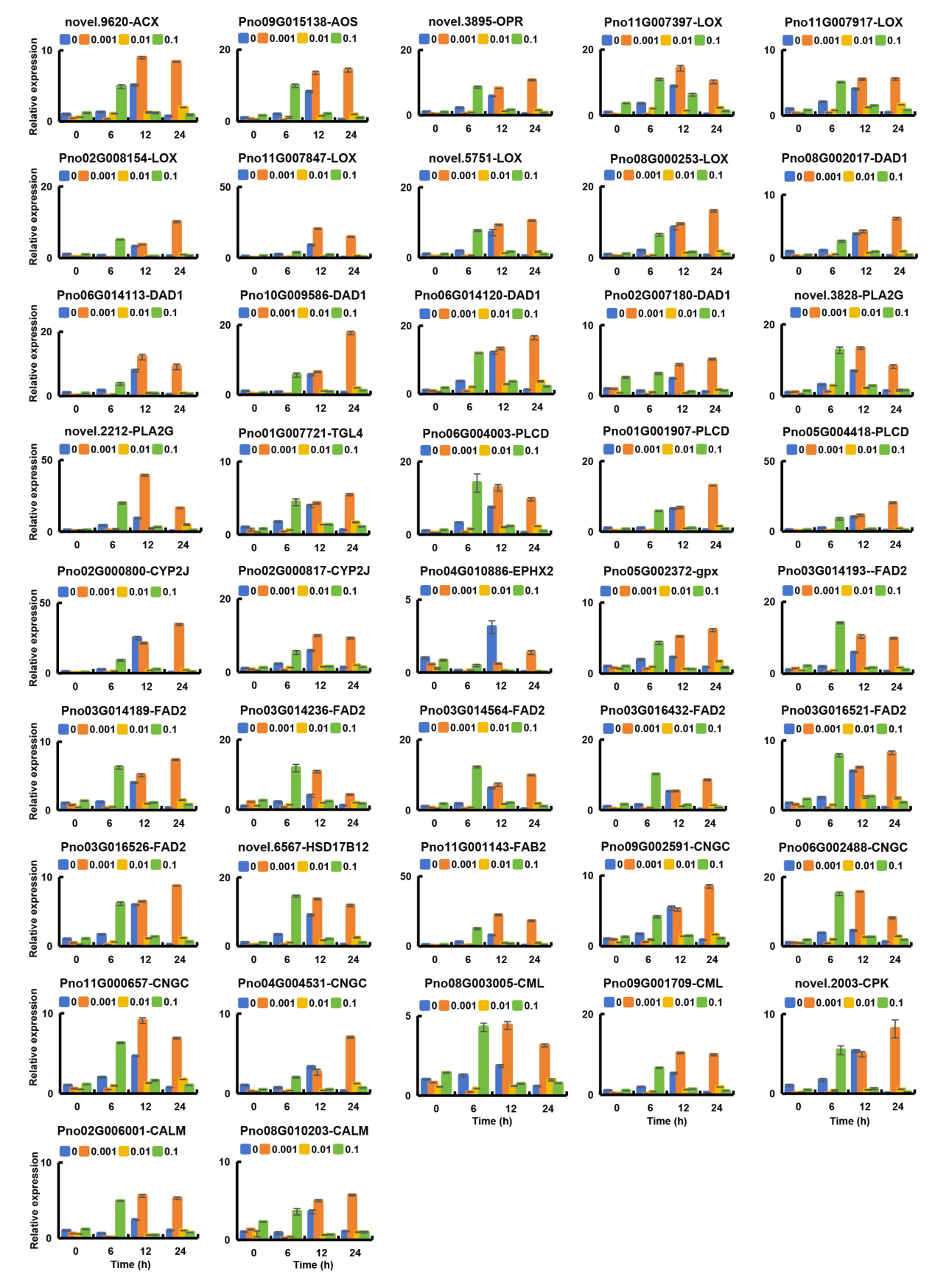


Figure S8 Key gene expression in *P. notoginseng*’s fibrous roots activated by exogenous JA application, as determined by qRT-PCR analysis. The data are expressed as the mean ± SEM (n = 3). ACX: acyl-CoA oxidase; AOS: hydroperoxide dehydratase; OPR: 12-oxophytodienoic acid reductase; LOX: lipoxygenase; DAD1: phospholipase A1; PLA2G: secretory phospholipase A2; TGL4: TAG lipase / steryl ester hydrolase / phospholipase A2 / LPA acyltransferase; PLCD: phosphatidylinositol phospholipase C; CYP2J: cytochrome P450 family 2 subfamily J; EPHX2: soluble epoxide hydrolase / lipid-phosphate phosphatase; gpx: glutathione peroxidase; FAD2: omega-6 fatty acid desaturase / acyl-lipid omega-6 desaturase (Delta-12 desaturase); HSD17B12: 17 beta-estradiol 17-dehydrogenase / very-long-chain 3-oxoacyl-CoA reductase; FAB2: acyl-[acyl-carrier-protein] desaturase; CNGC: cyclic nucleotide gated channel; CML: calcium-binding protein; CPK: calcium-dependent protein kinase; CALM: calmodulin.


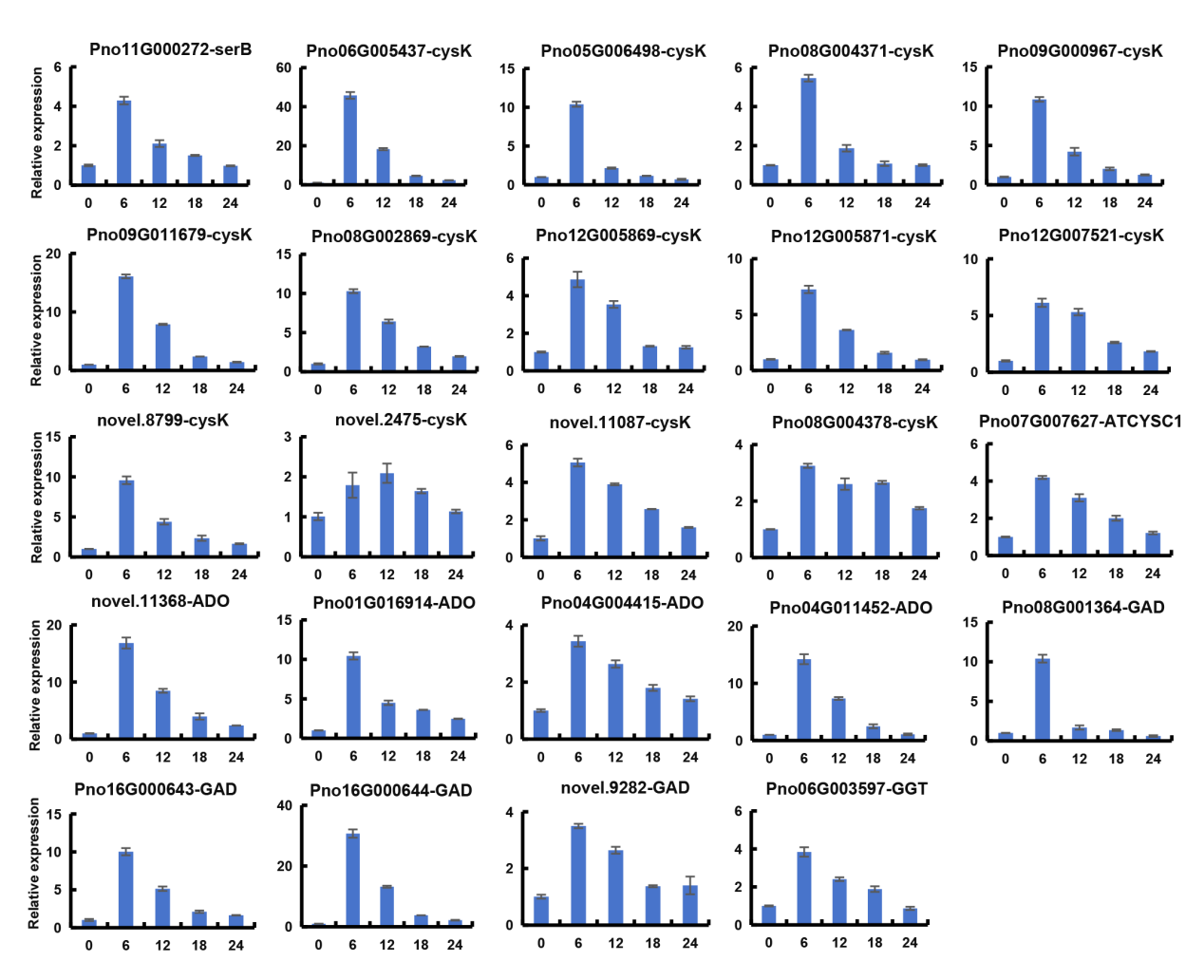


Figure S9 Key gene expression of taurine and hypotaurine metabolism in *P. notoginseng*’s fibrous roots activated by foliar *Alternaria panax* infection, as determined by qRT-PCR analysis. The data are expressed as the mean ± SEM (n = 3). serB: phosphoserine phosphatase; cysK: cysteine synthase; ATCYSC1: L-3-cyanoalanine synthase/ cysteine synthase; ADO: cysteamine dioxygenase; GGT: gamma-glutamyltranspeptidase / glutathione hydrolase / leukotriene-C4 hydrolase; GAD: glutamate decarboxylase.


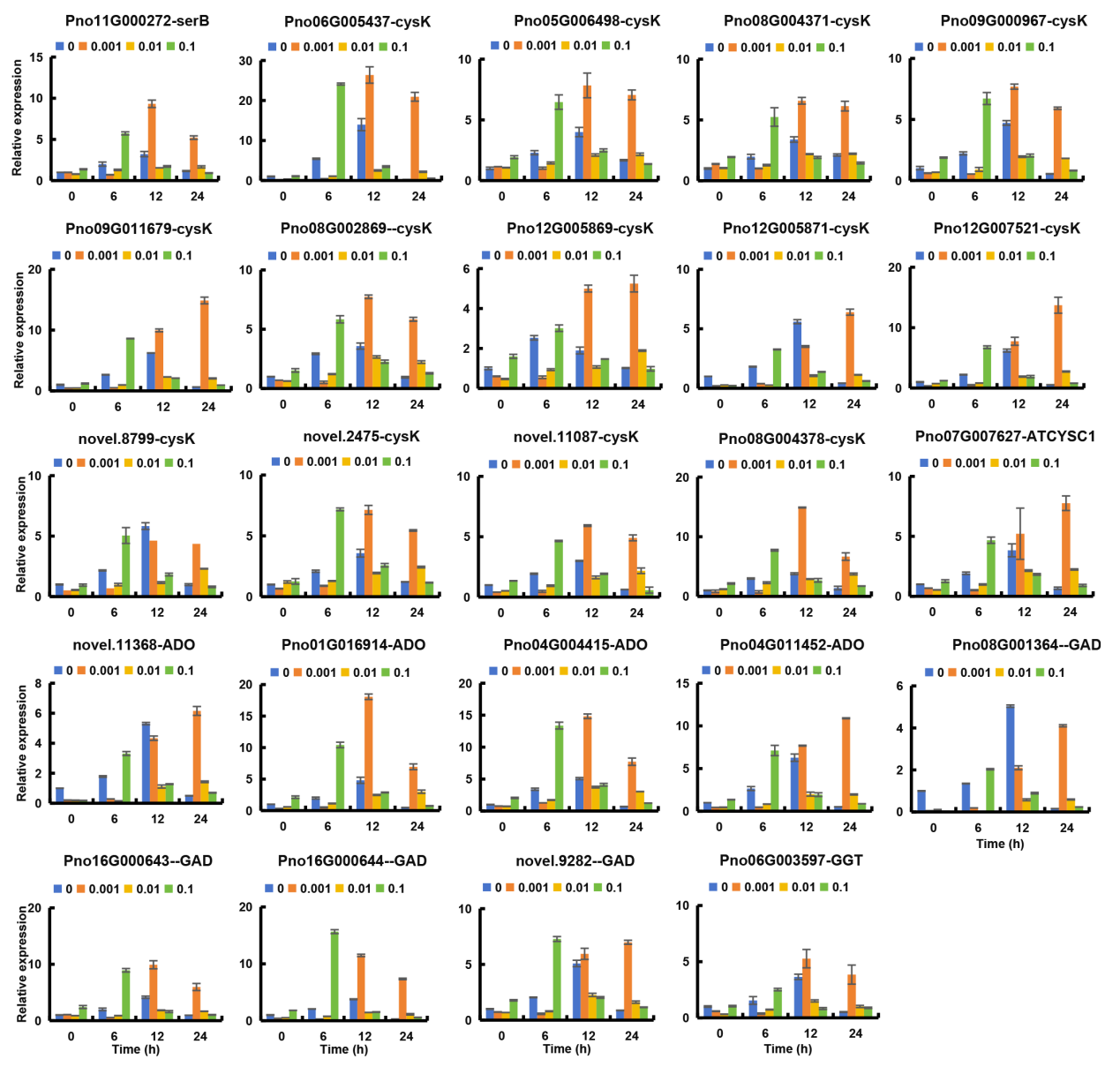


Figure S10 Key gene expression of taurine and hypotaurine metabolism in *P. notoginseng*’s fibrous roots activated by exogenous JA application, as determined by qRT-PCR analysis. The data are expressed as the mean ± SEM (n = 3). serB: phosphoserine phosphatase; cysK: cysteine synthase; ATCYSC1: L-3-cyanoalanine synthase/ cysteine synthase; ADO: cysteamine dioxygenase; GGT: gamma-glutamyltranspeptidase / glutathione hydrolase / leukotriene-C4 hydrolase; GAD: glutamate decarboxylase.


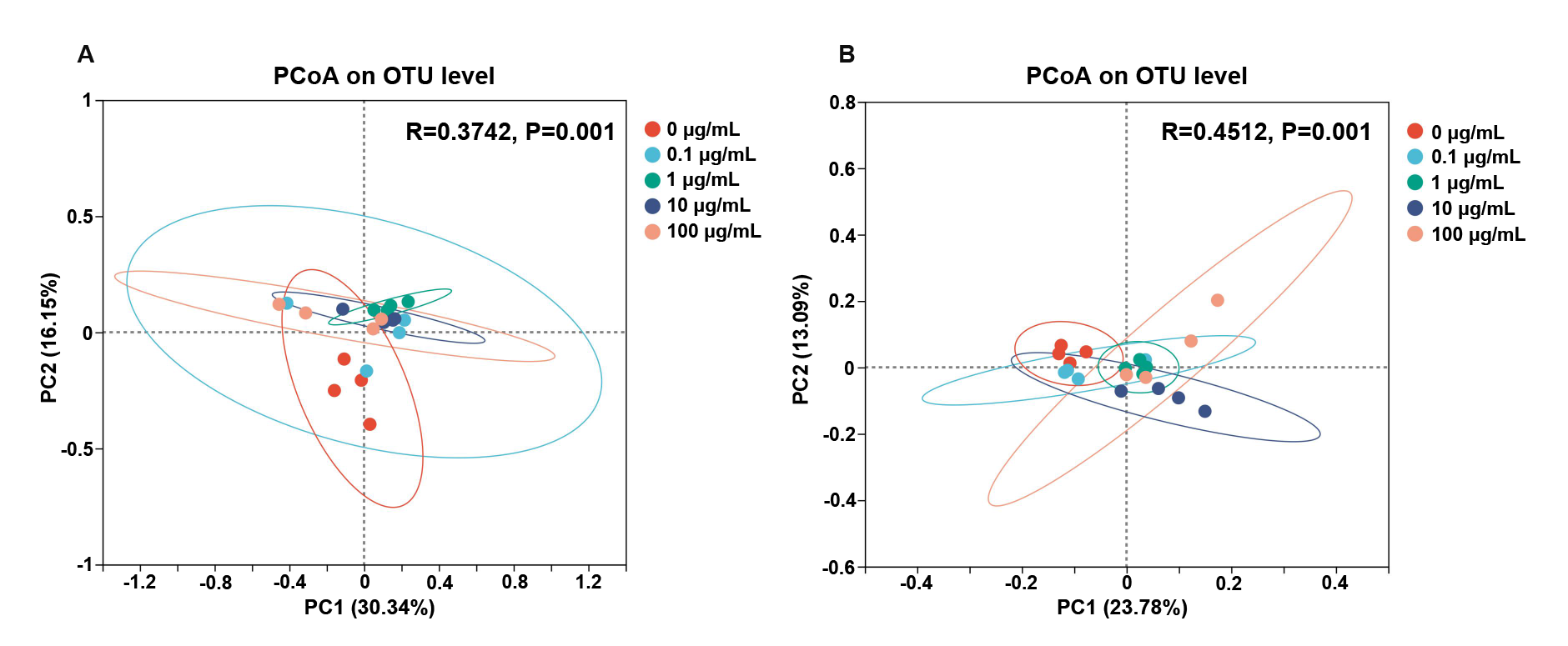


**Figure S11** PCoA analysis of fungi (A) and bacteria (B) in the rhizosphere soil following soil drenching with 2-aminoethanesulfonic acid.
