# Supplementary material for: Diversified alternaria pathogenicity alters plant–soil feedbacks through leaf–root-microbiome dynamics in agroforestry systems
Source: Hortic Res. 2025 May 21;12(8):uhaf137. doi: 10.1093/hr/uhaf137 (PMC12282122; doi:10.1093/hr/uhaf137)
Supplement: Web_Material_uhaf137 [file web_material_uhaf137.zip › Supplementary files.docx]

**Appendix S1. Evaluation the growth of *Panax notoginseng* in soil conditioned by Alternaria leaf spot in agroforestry system.**

In this study, mild and severe leaf spot symptoms were characterized by partial defoliation and complete leaf loss, respectively (Fig. 1A). Healthy *P. notoginseng* plants served as control (Fig. 1A). The remaining soil attached to the roots was collected and defined as rhizosphere soil^1^. These infection-conditioned rhizosphere soils of three-year-old *P. notoginseng* from agroforestry systems were used to assess the survival state of subsequent *P. notoginseng* according to previous method with some modification^2^. Briefly, 90 g (90%, w/w) of the sterilized normal soil was mixed with 10 g (10%, w/w) of the rhizosphere soil in a glass bottle. The soil in the glass bottle was thoroughly mixed. Ten healthy *P. notoginseng* seeds, sterilized with 1% sodium hypochlorite solution, were sown into the soil. Each treatment contained seven glass bottles. The glass bottles were placed in a greenhouse (25 ± 2◦C, 12 h light/12 h dark). The survival rate and dry weight were evaluated.

**Appendix S2. Pathogenicity determination of *Alternaria panax.***

The pathogenicity of five isolates of *A. panax* was determined on the leaves of *P. notoginseng* in *vitro*. The isolates were transferred to potato dextrose agar (PDA) and incubated for 5 days. A 6 mm agar plug was inoculated onto a pre-made wound on leaves, with a noncolonized agar plug used as control. The inoculated *P. notoginseng* leaves were placed in glass petri dishes lined with wet filter paper to maintain humidity. All petri dishes were kept in the dark at a temperature of 25 ± 2°C. Once the leaves showed symptomatic lesions, pathogens were isolated again. The lesion areas were scanned by an Epson perfection V850 Pro scanner, and their size was estimated using the measuring tool in Adobe Photoshop CS6.

Three strains, which displayed significant differences in lesion area, were selected for in *vivo* inoculation on one-year-old *P. notoginseng*. Each seedling was inoculated with two mycelial blocks. Each pot contained 8 seedlings, and all pots were placed in transparent boxes and incubated with a photoperiod of 16 h light/8 h dark at 25±2°C, with humidity maintained at levels conducive to pathogen infection. When the leaves showed symptomatic lesions, the lesion areas were scanned by an Epson perfection V850 Pro scanner, and their size was estimated using the measuring tool in Adobe Photoshop CS6.

**Appendix S3. Effects of different pathogenicity levels or intensity of foliar infection by *A. panax* on rhizospheric microbe community.**

Soil genomic DNA was extracted using E.Z.N.A.® soil DNA kit (Omega Bio-tek, Norcross, GA, USA) following the manufacturer’s instructions. Fungal ITS and bacterial 16S rRNA genes in the soil total DNA samples were sequenced using the Illumina Miseq PE300 platform (Illumina, San Diego, USA) according to the standard protocols by Majorbio Bio-Pharm Technology Co. Ltd. (Shanghai, China). The raw gene sequencing reads were demultiplexed, quality-filtered by fastp version 0.20.0^3^ and merged by FLASH version 1.2.7^4^. Operational taxonomic units (OTUs) were defined at ≥97% sequence identity using UPARSE version 7.1^5^. The taxonomic identities were determined according to the Unite (fungi) and Silva (bacteria) databases^6^. The data of each sample were then normalized according to the minimum data in the sample. Finally, PCoA, and community structure were analyzed on the majorbio platform (https://cloud.majorbio.com). Advanced random forest was performed using the OmicStudio tools at <https://www.omicstudio.cn/tool.>

**Appendix S4. Function evaluation of the microorganisms modified by foliar infection by *A. panax.***

The pathogenicity of *Ilyonectria* isolate on *P. notoginseng* roots was determined *in vitro* according to Luo’s method^7^. Thirty roots were inoculated with a noncolonized agar block as a control. After 5 days of inoculation, pathogens were reisolated from roots with symptomatic lesions. The antagonistic activity of differential bacteria *Pseudoxanthomonas* sp., *Mycobacterium* sp., *Pseudomonas aeruginosa*, *Rhodococcus* sp., and *Microbacterium oxydans* against *Ilyonectria* sp. was tested in a dual culture following the method described in a previous study^8^. These bacteria were isolated from the rhizosphere of *P. notoginseng*. *Rhodococcus* sp. and *Microbacterium oxydans* were mixed in equal proportions to test their synergistically antagonistic effects against *Ilyonectria* sp*.* The mycelial growth of the pathogen was determined by measuring the colony semidiameter. The growth inhibition rate was calculated as follows:

Growth inhibition rate (%) = 100 × (radial growth of control−radial growth of treated sample) / radial growth of control.

**Appendix S5. Transcriptome sequencing and data analysis of leaves and fibrous roots after different intensities of foliar infection by *A. panax.***

The total RNA was extracted from each leaf or root sample stored at -80°C using TRIzol reagent (Invitrogen, Carlsbad, CA, USA) and treated with DNase I (Fermentas, Carlsbad, CA, USA) according to the manufacturer’s instructions. A total of 1 μg of RNA per sample was used as input material for the RNA sample preparations. PCR products were purified (AMPure XP system) and library quality was assessed on the Agilent Bioanalyzer 2100 system. The library preparations were sequenced on an Illumina Hiseq platform. The obtained raw reads were cleaned using fastp v 0.19.3 by removing adapter sequences, reads containing poly-N more than 10%, and low-quality sequences (the number of low-quality (Q<=20) bases contained in reads exceeds 50% of the bases of the reads,). Clean reads were mapped to the *P. notoginseng* reference genome available at <http://herbalplant.ynau.edu.cn/>^9^. All the assembled reads were then annotated by employing the NCBI NR, GO, KEGG, Pfam, KOG, Tremble, and Swiss-Prot databases. False discovery rate (FDR) was used to determine the *p*-value threshold in multiple tests^10^. The absolute value of the log_2_ (fold change with FPKM) ≥ 1 and FDR < 0.05 were used as the threshold to determine significant differences in gene expression in this study. The deferentially expressed genes (DEGs) were analyzed by principal component analysis (PCA) and mapping to KEGG pathways.

**Appendix S6. UPLC-MS/MS analyses of leaves, stems, fibrous roots and root exudates after different intensities of foliar infection by *A. panax.***

Metabolome analysis was conducted by MetWare Biological Science and Technology Co. Ltd. (Wuhan, China). Briefly, 100 mg samples from leaves, stems, fibrous roots, or root exudates were extracted with 700 μL 70% aqueous methanol and vortex for 3 min and then centrifuged (12000 rpm, 4°C) for 10 min. The supernatant was filtered through a 0.22 μm microporous membrane prior to analysis on an ultra-performance liquid chromatography‒electrospray ionization tandem mass spectrometry (UPLC-ESI-MS/MS) system (UPLC, SHIMADZU Nexera X2; MS, Applied Biosystems 4500 QTRAP). The UPLC conditions were as follows: the column (Agilent SB-C18 1.8 µm, 2.1 mm×100 mm) temperature was 40 ℃, the injection volume was 4 μL, and the flow rate was 0.35 mL/min. The solvent system included mobile phase A (pure water with 0.1% formic acid) and mobile phase B (acetonitrile with 0.1% formic acid). A multistep gradient (0 min of 5% solution B, 0-9 min of 5%-95% solution B, 9-10 min of 95% solution B, 10.0-11.1 min of 95%-5% solution B, 11.1-14.0 min of 5% solution B) was used for all separations. LIT and triple quadrupole (QQQ) scans were acquired on a triple quadrupole-linear ion trap mass spectrometer (Q TRAP), AB4500 Q TRAP UPLC/MS/MS System, equipped with an ESI Turbo Ion-Spray interface, operating in positive and negative ion mode, and controlled by Analyst 1.6.3 software (AB Sciex). Metabolite profiling was carried out using a widely targeted metabolome method. Metabolites were quantified using a multiple reaction monitoring (MRM) method. Differential metabolites (DAMs) were screened for variable importance of the projection (VIP) ≥ 1 and |log_2_(fold change) | ≥ 1, followed by principal component analysis (PCA) and mapped to metabolic pathways in the KEGG compound database.

**Appendix S7. Determination of phytohormones content in *P. notoginseng.***

Phytohormones were quantified according to a method described previously^11^. Briefly, approximately 150 mg of *P. notoginseng* leaves after foliar infection for 24h was extracted with 1 mL of ice-cold ethyl acetate spiked with the internal standards (200 ng of D_6_-jasmonic acid, ^13^C_6_-jasmonic acid-eisoleucine conjugate, D_6_-abscisic acid, and D_5_-Indole-3-acetic acid, respectively). After centrifugation, the supernatants were next dried in a vacuum concentrator (Eppendorf). The pellets were each extracted with 0.2 mL of 50% (v/v) methanol. After centrifugation, the supernatants were injected into an UPLC-MS/MS system (LCMS-8040 system, Shimadzu) to obtain the peak areas of internal standards and those of the respective target compounds for determination of the concentrations of phytohormones.

**Appendix S8. Validation of gene expression by Quantitative Real-time PCR (qRT-PCR).**

qRT-PCR was carried out to validate the expression of DEGs in fibrous roots after foliar infection with 2 leaves by *A. panax* for 24 h. The total RNA was extracted using the TRIzol Kit (Promega, Fitchburg, WI), and complementary DNA (cDNA) was synthesized with a reverse transcript kit according to the manufacturer's instructions (Monad, Wuhan, China). qRT-PCR amplification was performed in a 10 μL final volume containing 1 μL of cDNA templates, 5 μL 2x SYBR Green PCR Master Mix, 0.7 μL of forward primer (0.7 μM), 0.7 μL of reverse primer (0.7 μM), and 2.6 μL of RNase-free water. qRT-PCR amplification had an initial heat activation of 95 °C for 2 min, followed by 40 cycles of 95 °C denaturation for 5 s, and 60 °C annealing for 30 s. The primers used were listed in Table S7. The relative expression of genes was calculated using the 2^−ΔΔCT^ method^12^, using 18 s rRNA as the internal standard. There were three replicates for each treatment.

**Appendix S9. Quantification of differential metabolites in fibrous root and root exudates.**

Based on the metabolome analysis, three significantly changed metabolites (arachidonic acid, 15(S) -Hydroxy-5z,8Z,11Z, 13e-eicosatetraenoic acid (15(S)-HETE), and 2-aminoethanesulfonic acid) were further selected to determine their presence and concentrations in fibrous roots and root exudates by gas chromatography (GC, Agilent HPLC-1100) and UPLC (Agilent GC7890) (Norminkoda Biotechnology Co., Ltd., Wuhan, China). The analysis of arachidonic acid and 15(S)-HETE utilized a DB-FFAP column (30 mm×0.25 mm, Agilent) with flame ionization detector (FID). The initial injection volume was 1 μL and the inlet temperature was 220 ℃. Back detector temperature was 270 ℃ and a flow rate was 1.2 mL/min. The analysis of 2-aminoethanesulfonic acid utilized a C18 column (4.6 mm×250 mm, 0.5 μm, Agilent). The solvent system included mobile phase A (1 mmol/L sodium acetate, pH=5.3) and mobile phase B (methanol: water =1: 1 (V: V)). Isocratic elution was used for all the separations with an initial injection volume of 20 μL and a flow rate of 1 mL/min. The column temperature was maintained at 38 ℃ and the wavelength is 360 nm. The column temperature was maintained at 38 ℃ and the wavelength is 360 nm. The target compounds in fibrous root and root exudates were identified by comparing their retention times to those of authentic standards arachidonic acid (CA: 506-32-1, purity ≥ 98%), 15(S)-HETE (CAS: 54845-95-3, purity ≥ 98%) and 2-aminoethanesulfonic acid (CAS: 107-35-7, purity ≥ 98%). The content of the target compounds in fibrous root and root exudates was quantified using standard curves that showed the linear relationship between the peak areas and the concentrations (Table S8).

**Appendix S10. Functional evaluation of arachidonic acid, 15(S)-HETE, and 2-aminoethanesulfonic acid on *Ilyonectria* sp.**

The effect of arachidonic acid, 15(S)-HETE, and 2-aminoethanesulfonic acid on the growth of *Ilyonectria* sp., was determined by the colony diameter method^13^. Briefly, a pure mycelium block (6 mm diameter) was placed in the middle of medium amended with arachidonic acid, 15(S)-HETE (final concentrations were 0, 0.0001, 0.001, 0.01, 0.1, and 1 μg/mL) or 2-aminoethanesulfonic acid (final concentrations were 0, 0.01, 0.1, 1, and 10 μg/mL). The mycelial growth of *Ilyonectria* sp. was determined by measuring the colony diameter after dark incubation at 25 °C for 7 days.

**Appendix S11. Effects of 2-aminoethanesulfonic acid on PSF and rhizospheric microbiome.**

Eight healthy seedlings were planted in each pot containing soil that had been continuously cultivated with *P. notoginseng.* This soil naturally harbors various soil-borne pathogens such as *Ilyonectria* spp., *Fusarium* spp., and *Monographella* spp.^7^ After 7 days, 50 mL of 2-aminoethanesulfonic acid, with final concentrations ranging from 0.01 to 100 μg/mL, was added to each pot. The plants were then incubated under a photoperiod of 16 h light/8 h dark at 25±2°C for 6 weeks. Sterilized water was used as a control. Each treatment contained eight pots. The survival rate and feedback ratio, incidence of root rot, fresh biomass of *P. notoginseng* were recorded and calculated. Following this, the composition and function of microbial communities in the rhizosphere soil of *P. notoginseng* were analyzed using the same methods as those employed for studying the effects of foliar infection by *A. panax*.

**Appendix S12. Functional evaluation of 2-aminoethanesulfonic acid on growth of the core beneficial microbes.**

To verify the function of 2-aminoethanesulfonic acid on microbes co-enriched in foliar infection by *A. panax* and treatments with 2-aminoethanesulfonic acid, the effects of 2-aminoethanesulfonic acid on the growth of the core beneficial microbes *Microbacterium oxydans* and *Rhodococcus* sp. were measured following a published procedure^13^. Briefly, bacterial isolates were cultured in beef extract peptone medium (NA) liquid medium for 24 h. The inoculum was prepared by centrifugation to eliminate the supernatant, followed by vortexing in a new NA medium to achieve a concentration of 1×10^6^ CFU/mL. Subsequently, 2-aminoethanesulfonic acid was added to the suspension, resulting in final concentrations of 0, 0.1, 1, 10 and 100 μg/mL. After a shaking incubation period for 24 h at 28 °C and 200 rpm, the suspensions were transferred to 96-well microplates and measured at 600 nm to determine proliferation (OD_600_ reads) with a Versa Max microplate reader (Molecular Devices, Sunnyvale, CA, United States). Six replicates of each treatment were included.

**Appendix S13. Functional verification of the core beneficial microbes on promoting plant growth.**

To determine the effect of *Microbacterium oxydans* and *Rhodococcus* sp. on the growth of *P. notoginseng*, ten surface-sterilized seeds were sown in each pot. The pots contained natural soil with no history of *P. notoginseng* cultivation, which had been sterilized by steaming at 90°C for 20 minutes. After growing the plants in the greenhouse for six months, fifty milliliters of *Microbacterium oxydans* or *Rhodococcus* sp. suspension (10^6^ CFU/mL) was inoculated into the rhizosphere soil of *P. notoginseng,* respectively. Pots treated with sterilized water were used as the blank control. Each treatment contained six replicates. Two months after treatment, the survival rate, incidence of root rot, fresh and dry biomass of *P. notoginseng* were recorded and calculated.

**Reference**

Guo C, Yang M, Jiang B. *et al*. Moisture controls the suppression of *Panax notoginseng* root rot disease by indigenous bacterial communities. *mSystems*. 2022; **7**:e0041822.

Mendes R, Kruijt M, de Bruijn I. *et al*. Deciphering the rhizosphere microbiome for disease-suppressive bacteria. *Science*. 2011; **332**:1097-1100.

Chen S, Zhou Y, Chen Y. *et al*. Fastp: an ultra-fast all-in-one FASTQ preprocessor. *Bioinformatics*. 2018; **34**:i884-90.

Magoč T, Salzberg SL. FLASH: fast length adjustment of short reads to improve genome assemblies. *Bioinformatics*. 2011; **27**:2957‐63.

Edgar RC. UPARSE: highly accurate OTU sequences from microbial amplicon reads. *Nat Methods*. 2013; **10**:996‐8.

Quast C, Pruesse E, Yilmaz P. The SILVA ribosomal RNA gene database project: improved data processing and web-based tools. *Nucleic Acids Res.* 2013; **41**:D590-6.

Luo L, Guo C, Wang L. *et al*. Negative plant-soil feedback driven by re-assemblage of the rhizosphere microbiome with the growth of *Panax notoginseng*. *Front Microbiol*. 2019; **10**:1597.

Sun WM, Ma YN, Yin YJ. *et al*. Effects of essential oils from zingiberaceae plants on root-rot disease of *Panax notoginseng*. *Molecules*. 2018; **23**:1021.

Yang Z, Liu G, Zhang G. *et al*. The chromosome-scale high-quality genome assembly of *Panax notoginseng* provides insight into dencichine biosynthesis. *Plant Biotechnol J*. 2021; **19**:869-71.

Reiner A, Yekutieli D, Benjamini Y. Identifying differentially expressed genes using false discovery rate controlling procedures. *Bioinformatics.* 2003; **19**: 368-75.

Qi J, Sun G, Wang L. *et al*. Oral secretions from *Mythimna separata* insects specifically induce defence responses in maize as revealed by high-dimensional biological data. *Plant Cell Environ*. 2016; **39**:1749-66.

Livak KJ, Schmittgen TD. Analysis of relative gene expression data using real-time quantitative PCR and the 2^−ΔΔCT^ method. *Methods*. 2001; **25**:402-8.

Luo L, Zhang J, Ye C. *et al*. Foliar pathogen infection manipulates soil health through root exudate-modified rhizosphere microbiome. *Microbiol Spectr*. 2022; **10**:e0241822.
